# A Residual-Driven ResCompFormer for Multi-Sensor Systematic Error Compensation and Target Trajectory Reconstruction

**DOI:** 10.3390/s26165199

**Published:** 2026-08-17

**Authors:** Sihua Wang, Jiongqi Wang, Bingxin Peng, Zhangming He, Xuanying Zhou

**Affiliations:** College of Science, National University of Defense Technology, Fuyuan Road No. 1, Changsha 410072, China; siwa_zz@163.com (S.W.); wjq_gfkd@163.com (J.W.); pengbingxinnudt@nudt.edu.cn (B.P.); hzmnudt@163.com (Z.H.)

**Keywords:** multi-sensor data fusion, systematic error, EMBET, B-spline, ResCompFormer

## Abstract

Multi-sensor data fusion is essential for accurate target tracking and trajectory reconstruction. However, common forms of systematic error in multi-sensor observations, including constant biases, linear drifts, and saturating exponential drifts, can degrade measurement consistency and trajectory estimation accuracy. Within the B-spline-constrained Error Model Best Estimate of Trajectory (EMBET) framework, B-spline coefficients and systematic-error parameters may produce similar observation responses, allowing part of the systematic-error response to be absorbed into the spline-coefficient correction and thereby weakening the identifiability of the systematic-error parameters. To avoid the weak-identifiability mechanism associated with the joint parametric estimation of trajectory and systematic-error terms, a residual-driven ResCompFormer method is proposed for systematic-error compensation and target trajectory reconstruction. First, a B-spline-constrained EMBET model is established to analyze the coupling between B-spline coefficients and systematic-error parameters. Systematic-error estimation is then removed from the joint EMBET parameter-estimation problem and reformulated as observation-domain error-sequence prediction, and ResCompFormer is employed to capture temporal dependencies and cross-channel correlations in multi-sensor residuals. The predicted errors are fed back to correct the observations, followed by iterative trajectory re-estimation. Simulation results confirm the systematic-error absorption mechanism and show that the proposed method outperforms the considered model-driven and data-driven methods in both systematic-error compensation and trajectory reconstruction, including iterative and stepwise EMBET variants. Additional experiments demonstrate the robustness of the proposed method to variations in systematic-error characteristics and sensor availability.

## 1. Introduction

Accurate trajectory estimation is essential for performance evaluation, guidance and control analysis, and measurement-system assessment in aerospace flight-test and range-testing campaigns [1,2]. These tasks commonly involve long observation intervals, complex environments, incomplete coverage by individual instruments, and heterogeneous measurement accuracies. A single sensor is therefore unlikely to provide uniformly reliable trajectory information over an entire flight arc. Radar, electro-optical theodolites, telemetry systems, and other heterogeneous sensors are commonly used to observe the same target cooperatively [3,4]. Recent studies on integrated and tightly coupled trajectory estimation have shown that fusing heterogeneous observations within a unified estimation framework better exploits complementary information and provides redundant constraints for trajectory reconstruction and sensor-error calibration [5,6].

Multi-sensor measurements generally contain both random and systematic errors. Random errors mainly arise from sensor noise, environmental disturbances, and other stochastic uncertainties and are commonly modeled as zero-mean random noise with a specified covariance structure. Systematic errors originate from persistent imperfections in the measurement and data-processing chain, such as axis misalignment, zero offsets, ranging biases, time-synchronization mismatch, coordinate-reference inconsistencies, and variations in sensor operating conditions. In the observation domain, these errors may appear as constant biases, slowly varying drifts, nonlinear time-varying drifts, or combinations of several components [7,8]. When propagated through the multi-sensor fusion model, such structured errors may produce persistent trajectory deviations or local distortions in the reconstructed trajectory [9]. Moreover, different sensors and observation channels may contain systematic errors with distinct amplitudes and temporal characteristics. Inadequate identification or compensation of these errors reduces the consistency among heterogeneous observations and degrades multi-sensor fusion accuracy [10,11]. Therefore, systematic error modeling, parameter identifiability analysis, and error compensation remain important issues in multi-sensor trajectory determination.

The Error Model Best Estimate of Trajectory (EMBET) method is a model-driven approach for the post-processing of range-measurement data. It represents the target trajectory and systematic-error parameters in a unified nonlinear model and estimates them jointly by exploiting redundant observations from multiple sensors and observation channels [8,12]. Iterative error identification and clustering-assisted identification were introduced to improve error separation under complex measurement conditions [9,13]. These approaches retain explicit physical interpretations and are compatible with established range-data processing procedures. Their performance, however, depends on the trajectory parameterization, the prescribed systematic error model, and the observation geometry. Model mismatch, numerical ill-conditioning, and weak identifiability may arise when the true error pattern differs from the assumed model or when trajectory corrections and systematic error perturbations generate similar responses in the observation space.

In a conventional EMBET formulation without trajectory parameterization, treating the target state at every epoch as an independent unknown for long observation arcs with high sampling rates produces a rapidly growing parameter vector, making the associated normal equations computationally expensive to form and solve. Reduced-parameter models address this problem by representing a continuous trajectory with splines, polynomials, or other low-dimensional basis functions [14,15,16]. B-splines are particularly suitable because of their local support, smoothness, differentiability, and flexibility for nonuniform sampling. They have been used in heterogeneous measurement fusion, trajectory determination, and trajectory estimation with incomplete observations [17,18,19]. Continuous-time and batch parameterizations have also been adopted to integrate observations from global navigation satellite systems, inertial sensors, imaging sensors, and laser scanners [5,6]. Recent work further emphasizes continuous-time B-spline trajectory representations for multi-sensor state estimation and calibration. A recent review systematically summarizes their applications in asynchronous multi-source fusion, offline calibration, and online odometry [20]. Continuous-time B-spline trajectories have also been adopted for camera–IMU extrinsic calibration and multimodal spatiotemporal calibration [21,22].

Reduced-order trajectory parameterization does not necessarily improve the identifiability of systematic-error parameters. In a linearized observation model, increments in the B-spline coefficients and systematic-error parameters are mapped into the observation space through their respective sensitivity matrices. When these column spaces are strongly correlated, the two parameter groups can produce similar observation responses, making it difficult to distinguish spline-coefficient corrections from systematic-error effects. In this study, parameter coupling refers specifically to the similarity between the observation responses induced by the B-spline coefficients and those induced by the systematic-error parameters. Under strong coupling, part of the observation variation caused by systematic errors can also be represented by changes in the B-spline coefficients. This overlap directly weakens systematic-error estimation and may consequently degrade trajectory reconstruction. Constant biases and slowly varying drifts are particularly likely to be confused with smooth, low-frequency trajectory corrections. Nonlinear drifts may also be approximated locally when the spline representation has sufficient flexibility. The estimability of systematic error states therefore depends on trajectory excitation, sensor configuration, observation type, and measurement sensitivity [23].

Existing studies have attempted to mitigate parameter coupling and weak identifiability through regularization, spline-structure adjustment, and stronger observation constraints. Sparse regularization encourages prominent sensor errors to be represented by explicit error terms rather than absorbed into trajectory corrections [7]. Other methods adjust the spline order, knot number, or knot locations, or introduce sparse B-spline representations to limit trajectory flexibility and alleviate ill-posed estimation [17,18,19]. These strategies can improve numerical stability, but they generally rely on prescribed error structures, parameter distributions, or regularization assumptions. A single parametric error model may be insufficient when heterogeneous observation channels contain systematic errors with different amplitudes and temporal patterns. Semiparametric studies similarly indicate that uncertain and complex systematic errors require a balance between physical modeling and adaptive representations [24].

Data-driven methods provide an alternative by learning nonstationary sensor-error patterns directly from multi-channel sequences. Long short-term memory networks, convolutional neural networks, and self-supervised models have been applied to inertial-sensor calibration, denoising, and navigation by learning bias, drift, and nonstationary characteristics from measurement sequences [25,26,27,28]. Recent work has further extended this direction toward online sensor self-calibration and adaptive compensation. Memory-efficient neural calibration has been developed for on-device adaptation of MEMS inertial sensors, while Transformer-assisted inertial localization has been used to adapt noise statistics and compensate long-term localization errors in the presence of missing IMU data [29,30]. Dynamic receptive-field mechanisms and Transformer architectures can further capture long-range temporal dependencies and nonlinear variations [31,32]. Although these studies primarily concern inertial sensors, the underlying sequence-modeling principle is also relevant to heterogeneous trajectory measurements: the error component may exhibit temporal continuity, channel-dependent behavior, and cross-channel dependence that cannot be adequately represented by a fixed low-order parametric model.

A practical limitation of supervised error-compensation learning is the need for paired measurement sequences and sufficiently accurate systematic-error labels, which may be difficult to obtain because the systematic component is not directly observable without an accurate reference trajectory or independent calibration. Recent temporal–contextual self-supervised and class-aware semi-supervised frameworks have shown that unlabeled time-series data can be exploited for representation learning before task-specific refinement [33,34]. Although developed for fault diagnosis rather than trajectory-error compensation, these approaches suggest a potential extension in which the residual encoder is pretrained on unlabeled multi-sensor sequences and then fine-tuned with systematic-error and trajectory-reconstruction objectives.

Residuals from an initial trajectory estimate contain random noise together with systematic components that have not been fully separated from the trajectory representation. Different systematic error forms produce distinct temporal structures. A constant bias produces a persistent offset, a linear drift produces a progressively changing residual, and a saturating exponential drift produces a smooth nonlinear transition toward an asymptotic level. In a heterogeneous sensor network, residual channels are also coupled through the common target motion, coordinate transformations, observation geometry, sensor identity, and measurement type. A compensation model should therefore capture both temporal dependencies and relationships among observation channels. Informer and Autoformer demonstrated the effectiveness of attention mechanisms for long-sequence modeling [35,36]. Recent developments include the patch-based and channel-independent representation of PatchTST [37], the variable-as-token design of iTransformer [38], and channel-wise attention in SAMformer [39]. Pretrained time-series models have also been explored for cross-domain zero-shot and probabilistic forecasting [40,41]. These strategies broaden long-sequence representation and multivariate time-series modeling, but they primarily target generic forecasting rather than signed systematic-error compensation coupled with trajectory reconstruction.

ResCompFormer retains the standard scaled dot-product attention mechanism [42] but reorganizes the input representation for systematic-error compensation. At each sampling epoch, numerical residuals are augmented with sensor-identity and measurement-type embeddings and jointly projected across channels into one latent token before temporal positional encoding and self-attention. Thus, time remains the attention axis while cross-channel information is fused before attention. This differs from the channel-independent temporal patching of PatchTST [37], the variable-as-token design of iTransformer [38], and the explicit cross-variable or channel-wise attention mechanisms used by Crossformer [43] and SAMformer [39]. A non-autoregressive decoder then predicts the complete multichannel signed systematic-error sequence in parallel.

The network output is further coupled to B-spline-constrained EMBET rather than treated as an isolated sequence prediction. During inference, systematic-error prediction and trajectory re-estimation are performed iteratively; during training, a differentiable EMBET layer propagates the trajectory-reconstruction objective through the re-estimation procedure to the network.

To address these limitations, this study proposes a residual-driven ResCompFormer framework for multi-sensor systematic-error compensation and target trajectory reconstruction. The main contributions are summarized as follows.

First, a B-spline-constrained EMBET model is established, and a weighted sensitivity-space analysis is developed to characterize how systematic-error responses are projected onto and absorbed into the spline sensitivity space. This analysis distinguishes structural non-identifiability from practical weak identifiability and clarifies the information that remains available for systematic-error estimation. Second, systematic-error estimation is removed from the joint parametric estimation of trajectory and systematic-error terms and reformulated as signed observation-domain sequence prediction. This decoupling avoids the weak-identifiability mechanism caused by direct competition between B-spline coefficients and systematic-error parameters within the same estimation problem. ResCompFormer then exploits same-epoch multichannel feature fusion, temporal self-attention, and parallel non-autoregressive decoding to predict the systematic-error sequence. Third, ResCompFormer is coupled to B-spline-constrained EMBET through iterative compensation and trajectory re-estimation, while a differentiable trajectory loss links observation-domain compensation to the final trajectory objective during training. Within this decoupled formulation, temporal and cross-channel regularities learned from the training data provide a data-driven prior for systematic-error prediction without altering the underlying structural identifiability conditions.

Simulations are conducted using a nonlinear six-degree-of-freedom fixed-wing unmanned aerial vehicle (UAV) model and a heterogeneous radar-electro-optical sensor network. Constant biases, linear drifts, and saturating exponential drifts are considered in the weak-identifiability and compensation experiments. The proposed method predicts observation-domain error sequences without explicitly fitting the corresponding parametric coefficients during inference. Section 2 presents the B-spline-constrained EMBET model and the sensitivity-space analysis. Section 3 describes ResCompFormer and the iterative compensation procedure. Section 4 presents the simulation design and experimental evaluation, and Section 5 concludes the paper. A summary of the principal mathematical notation used throughout the manuscript is provided in Appendix A.

## 2. B-Spline-Constrained EMBET Fusion Model and Parameter Estimation

### 2.1. Multi-Sensor Observation Model and B-Spline Trajectory Parameterization

Consider an aerial target observed by a heterogeneous multi-sensor system. Let the position and velocity of the target in the Earth-centered, Earth-fixed (ECEF) frame at time *t* be denoted by $x\left(t\right)={\left(x\left(t\right),y\left(t\right),z\left(t\right)\right)}^{T}$ and $\dot{x}\left(t\right)={\left(\dot{x}\left(t\right),\dot{y}\left(t\right),\dot{z}\left(t\right)\right)}^{T}$, respectively. The sensor measurements may include range, azimuth, elevation, and range rate, all expressed in a local east–north–up (ENU) frame centered at the corresponding sensor station. At time *t*, the true range $R_{i}\left(t\right)$, azimuth angle $A_{i}\left(t\right)$, elevation angle $E_{i}\left(t\right)$, and range rate ${\dot{R}}_{i}\left(t\right)$ of the target can be expressed as follows

(1) $$\left\{\begin{array}{c} R_{i}\left(t\right)=\sqrt{x_{s,i}^{2}\left(t\right)+y_{s,i}^{2}\left(t\right)+z_{s,i}^{2}\left(t\right)},\\ A_{i}\left(t\right)=atan2\left(x_{s,i}\left(t\right),y_{s,i}\left(t\right)\right),\\ E_{i}\left(t\right)=\arctan\left(\frac{z_{s,i}\left(t\right)}{\sqrt{x_{s,i}^{2}\left(t\right)+y_{s,i}^{2}\left(t\right)}}\right),\\ {\dot{R}}_{i}\left(t\right)=\frac{x_{s,i}\left(t\right){\dot{x}}_{s,i}\left(t\right)+y_{s,i}\left(t\right){\dot{y}}_{s,i}\left(t\right)+z_{s,i}\left(t\right){\dot{z}}_{s,i}\left(t\right)}{\sqrt{x_{s,i}^{2}\left(t\right)+y_{s,i}^{2}\left(t\right)+z_{s,i}^{2}\left(t\right)}}, \end{array}\right.$$

where $x_{s,i}\left(t\right)={\left(x_{s,i}\left(t\right),y_{s,i}\left(t\right),z_{s,i}\left(t\right)\right)}^{T}$ and ${\dot{x}}_{s,i}\left(t\right)={\left({\dot{x}}_{s,i}\left(t\right),{\dot{y}}_{s,i}\left(t\right),{\dot{z}}_{s,i}\left(t\right)\right)}^{T}$ denote the target position and velocity in the local ENU frame of sensor *i*. The components $x_{s,i}\left(t\right)$, $y_{s,i}\left(t\right)$, and $z_{s,i}\left(t\right)$ correspond to the east, north, and up directions. The azimuth angle is measured clockwise from local north. The target position and velocity relative to sensor *i* are transformed from the ECEF frame to the local ENU frame following the coordinate transformation in [44].

Let the target state at time *t* be denoted by $\alpha \left(t\right)={\left[x^{T}\left(t\right),{\dot{x}}^{T}\left(t\right)\right]}^{T}$. The corresponding noiseless measurements are nonlinear functions of the target state α(t). Let the sampling epochs be collected in the vector $t={\left(t_{1},t_{2},\ldots ,t_{m}\right)}^{T}$. The stacked target-state vector to be estimated is defined as $X={\left(\alpha {\left(t_{1}\right)}^{T},\alpha {\left(t_{2}\right)}^{T},\ldots ,\alpha {\left(t_{m}\right)}^{T}\right)}^{T}$.

Because different sensor types may provide different measurement components, define the set of candidate measurement types as $D=\{R,\dot{R},A,E\}$, and let $D_{i}\subseteq D$ denote the set of measurement types available from sensor *i*, where i=1,2,…,n. For sensor *i*, let $d\in D_{i}$ denote a generic available measurement type. The corresponding measurement vector over all sampling epochs is defined as

(2) $$y_{i,d}={\left[y_{i,d}\left(t_{1}\right),y_{i,d}\left(t_{2}\right),\ldots ,y_{i,d}\left(t_{m}\right)\right]}^{T}\in R^{m},$$

where $y_{i,d}\left(t_{j}\right)$ denotes the measurement of type *d* provided by sensor *i* at sampling epoch $t_{j}$.

The measurement vector of sensor *i*, obtained by stacking all available measurement components, is expressed as

(3) $$y_{i}={\left[y_{i,d_{1}}^{T},y_{i,d_{2}}^{T},\ldots ,y_{i,d_{|D_{i}|}}^{T}\right]}^{T}\in R^{m|D_{i}|},$$

where $d_{c}\in D_{i}$, $c=1,2,\ldots ,|D_{i}|$, denotes the *c*th measurement component available from sensor *i*, and $|D_{i}|$ denotes the number of measurement components provided by that sensor.

The measurement model for sensor *i* can then be written as

(4) $$y_{i}=f_{i}\left(X\right)+u_{i}\left(t;\gamma \right)+\epsilon_{i},\;y_{i}\in R^{m|D_{i}|},$$

where $f_{i}\left(X\right)\in R^{m|D_{i}|}$ denotes the nonlinear observation function for sensor *i*, stacked over all sampling epochs and available measurement components; $u_{i}\left(t;\gamma \right)\in R^{m|D_{i}|}$ denotes the corresponding systematic-error vector, stacked in the same order and parameterized by the systematic-error parameter vector $\gamma ={\left(\gamma_{1},\gamma_{2},\ldots ,\gamma_{n_{\gamma }}\right)}^{T}\in R^{n_{\gamma }}$; and $\epsilon_{i}\in R^{m|D_{i}|}$ denotes the random measurement-noise vector for sensor *i*, stacked according to the same ordering.

Stacking the measurement vectors from all sensors yields the global observation vector $Y={\left(y_{1}^{T},y_{2}^{T},\ldots ,y_{n}^{T}\right)}^{T}\in R^{mC}$, where $C=\sum_{i=1}^{n}\left|D_{i}\right|$ denotes the total number of available observation channels, with each channel corresponding to a sensor–measurement-component pair. The resulting joint nonlinear observation model is

**Figure 1:**
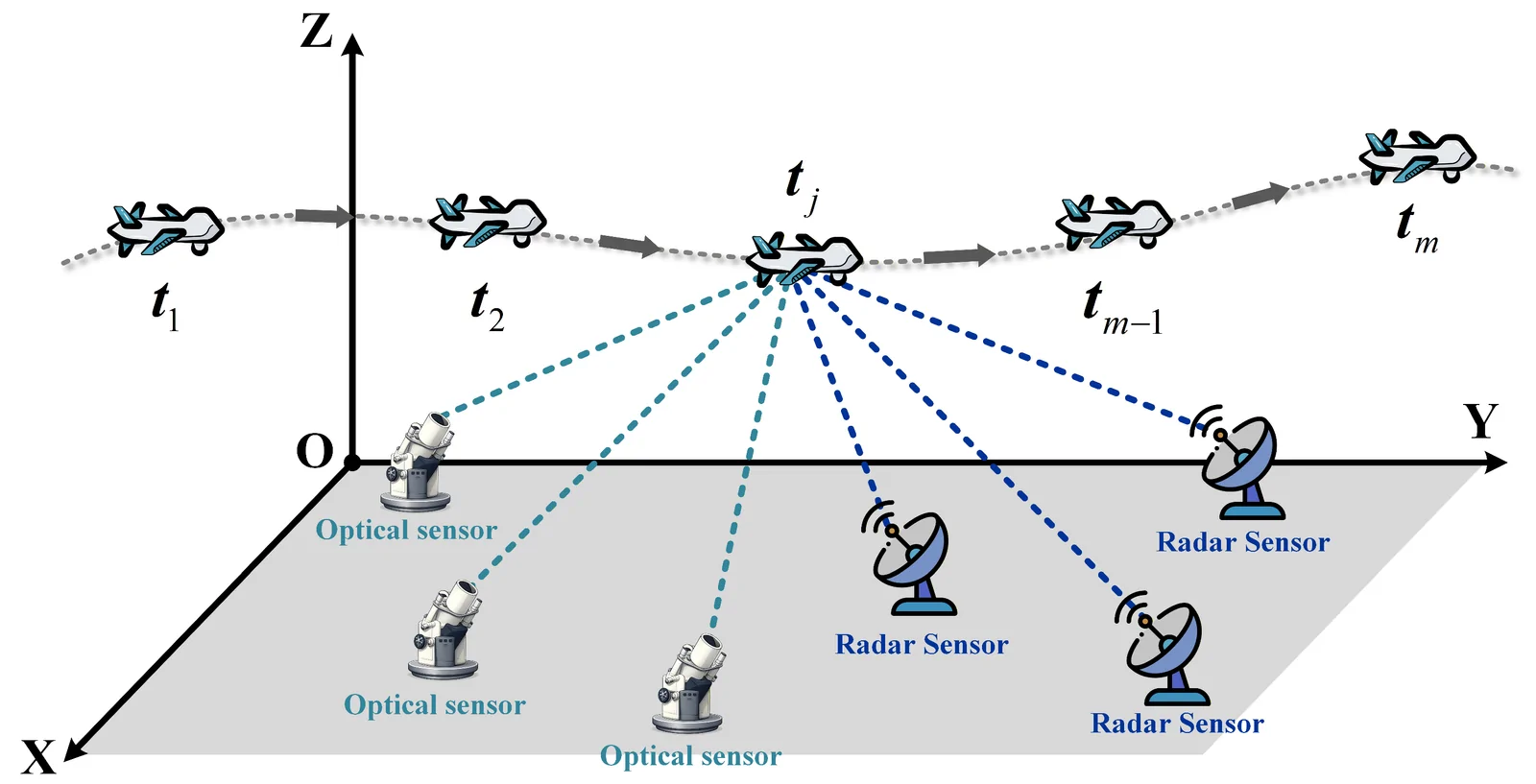
Schematic of the joint observation model for an aerial target observed by a heterogeneous multi-sensor system.

(5) Y=F(X)+U(t;γ)+ϵ,

where $F\left(X\right)={\left(f_{1}^{T}\left(X\right),f_{2}^{T}\left(X\right),\ldots ,f_{n}^{T}\left(X\right)\right)}^{T}\in R^{mC}$ denotes the stacked noiseless observation vector predicted from the trajectory X, $U\left(t;\gamma \right)={\left(u_{1}^{T}\left(t;\gamma \right),u_{2}^{T}\left(t;\gamma \right),\ldots ,u_{n}^{T}\left(t;\gamma \right)\right)}^{T}\in R^{mC}$ is the systematic-error vector, and $\epsilon ={\left(\epsilon_{1}^{T},\epsilon_{2}^{T},\ldots ,\epsilon_{n}^{T}\right)}^{T}\in R^{mC}$ is the random measurement-noise vector. Assume that ϵ has zero mean, i.e., E[ϵ]=0, and a positive definite covariance matrix $\mathrm{Cov}\left(\epsilon \right)=\Sigma \in R^{mC\times mC}$. Figure 1 illustrates the joint observation geometry between the heterogeneous multi-sensor system and the aerial target. The observations acquired by different sensor types at multiple sampling epochs are stacked into the global observation vector Y.

To reduce the trajectory dimensionality while retaining a smooth continuous-time representation, the target trajectory in the ECEF frame is parameterized using B-spline basis functions of order p+1. Let the sampling epochs be $t_{j}\in \left[a,b\right]$, j=1,…,m.

Consider the strictly increasing extended knot vector $T=\{T_{-p},\ldots ,T_{N+p+1}\}$, where $T_{0}=a$ and $T_{N+1}=b$, and *N* denotes the number of interior knots in (a,b). The N+2p+2 knots satisfy

(6) $$T_{-p}<\ldots <T_{0}<\ldots <T_{N+1}<\ldots <T_{N+p+1}.$$

The B-spline basis functions whose supports intersect [a,b] are indexed by τ=−p,…,N. Therefore, the total number of basis functions is q=N+p+1.

Let $B_{\tau ,p+1}\left(t\right)$ denote the B-spline basis function of order p+1. Define $w_{\tau ,p+1}\left(t\right)=\prod_{r=\tau }^{\tau +p+1}\left(t-T_{r}\right)$. Since the knots are distinct, $w_{\tau ,p+1}^{\prime }\left(T_{k}\right)\neq 0$. The basis function can then be expressed as

(7) $$B_{\tau ,p+1}\left(t\right)=\left(T_{\tau +p+1}-T_{\tau }\right)\sum_{k=\tau }^{\tau +p+1}\frac{{\left(T_{k}-t\right)}_{+}^{p}}{w_{\tau ,p+1}^{\prime }\left(T_{k}\right)},\;\tau =-p,\ldots ,N,$$

where ${\left(u\right)}_{+}^{p}=\max{\left(u,0\right)}^{p}$. Its first derivative is

(8) $${\dot{B}}_{\tau ,p+1}\left(t\right)=\frac{pB_{\tau ,p}\left(t\right)}{T_{\tau +p}-T_{\tau }}-\frac{pB_{\tau +1,p}\left(t\right)}{T_{\tau +p+1}-T_{\tau +1}},\;\tau =-p,\ldots ,N.$$

Define the basis vector and its derivative, respectively, as $B_{p+1}\left(t\right)=[B_{-p,p+1}\left(t\right),\ldots ,$$B_{N,p+1}\left(t\right)]\in R^{1\times q},$ and ${\dot{B}}_{p+1}\left(t\right)=\left[{\dot{B}}_{-p,p+1}\left(t\right),\ldots ,{\dot{B}}_{N,p+1}\left(t\right)\right]\in R^{1\times q}$.

Let $b_{x},b_{y},b_{z}\in R^{q}$ denote the spline coefficient vectors in the three coordinate directions, where, for example, $b_{x}={\left[b_{x,-p},\ldots ,b_{x,N}\right]}^{T}$. The target position and velocity are approximated by

(9) $$\left\{\begin{array}{c} x\left(t\right)\approx B_{p+1}\left(t\right)b_{x},\\ y\left(t\right)\approx B_{p+1}\left(t\right)b_{y},\\ z\left(t\right)\approx B_{p+1}\left(t\right)b_{z}, \end{array}\right.\;\left\{\begin{array}{c} \dot{x}\left(t\right)\approx {\dot{B}}_{p+1}\left(t\right)b_{x},\\ \dot{y}\left(t\right)\approx {\dot{B}}_{p+1}\left(t\right)b_{y},\\ \dot{z}\left(t\right)\approx {\dot{B}}_{p+1}\left(t\right)b_{z}. \end{array}\right.$$

Let $b={\left[b_{x}^{T},b_{y}^{T},b_{z}^{T}\right]}^{T}\in R^{3q}$ and $\alpha \left(t\right)={\left[x\left(t\right),y\left(t\right),z\left(t\right),\dot{x}\left(t\right),\dot{y}\left(t\right),\dot{z}\left(t\right)\right]}^{T}$. Then

(10) α(t)≈B(t)b,

where

(11) $$B\left(t\right)=\left[\begin{array}{ccc} B_{p+1}\left(t\right) & 0 & 0\\ 0 & B_{p+1}\left(t\right) & 0\\ 0 & 0 & B_{p+1}\left(t\right)\\ {\dot{B}}_{p+1}\left(t\right) & 0 & 0\\ 0 & {\dot{B}}_{p+1}\left(t\right) & 0\\ 0 & 0 & {\dot{B}}_{p+1}\left(t\right) \end{array}\right]\in R^{6\times 3q}.$$

By stacking the trajectory states at all sampling epochs, define

(12) $$B_{s}={\left[B^{T}\left(t_{1}\right),\ldots ,B^{T}\left(t_{m}\right)\right]}^{T},$$

such that $X\left(b\right)\approx B_{s}b$. Using this representation, (5) can be expressed in terms of the B-spline coefficient vector as

(13) Y=F(b)+U(t;γ)+ϵ,

where $F\left(b\right)≜F\left(X\left(b\right)\right)\approx F\left(B_{s}b\right)$.

The spline coefficient vector b and the systematic-error parameter vector γ are jointly estimated by solving

(14) $$\left(\hat{b},\hat{\gamma }\right)=\underset{b,\gamma }{arg min}{∥Y-F(b)-U(t;\gamma )∥}_{P}^{2},$$

where ${∥r∥}_{P}^{2}=r^{T}Pr$ and $P=\Sigma^{-1}$. The reconstructed trajectory and the estimated systematic-error vector are then given by

(15) $$\hat{X}=B_{s}\hat{b},\;\hat{U}\left(t\right)=U\left(t;\hat{\gamma }\right),$$

respectively.

### 2.2. Identifiability Analysis of the Coupling Between Spline Coefficients and Systematic Errors

The B-spline-constrained EMBET model represents the target trajectory using the spline coefficient vector b and parameterizes the systematic errors using γ. These two parameter groups are jointly estimated by minimizing the weighted nonlinear least-squares objective in (14).

B-spline parameterization reduces the number of trajectory variables and provides a smooth continuous-time trajectory representation. However, because the B-spline basis can represent smooth low-frequency variations, slowly varying systematic errors may be partially absorbed into the estimated trajectory, biasing the systematic-error parameter estimates. The coupling between the B-spline coefficients and the systematic-error parameters is examined by locally linearizing the observation model.

Let $\left(b_{k},\gamma_{k}\right)$ denote the current estimates of the spline coefficients and systematic-error parameters at iteration *k*. Performing a first-order Taylor expansion of (13) in the neighborhood of $\left(b_{k},\gamma_{k}\right)$ yields

(16) $$\begin{array}{cc} Y & \approx F\left(b_{k}\right)+U\left(t;\gamma_{k}\right)+\frac{\partial F}{\partial b}{|}_{b_{k}}\left(b-b_{k}\right)\\ \; & +\frac{\partial U}{\partial \gamma }{|}_{\gamma_{k}}\left(\gamma -\gamma_{k}\right)+\epsilon . \end{array}$$

Let $l_{k}=Y-F\left(b_{k}\right)-U\left(t;\gamma_{k}\right)$, $\Delta b_{k}=b-b_{k}$, and $\Delta \gamma_{k}=\gamma -\gamma_{k}$. In addition, define

(17) $$J_{b_{k}}=\frac{\partial F}{\partial b}{|}_{b_{k}},J_{\gamma_{k}}=\frac{\partial U}{\partial \gamma }{|}_{\gamma_{k}}.$$

Equation (16) can therefore be written in the following linearized form

(18) $$l_{k}=J_{b_{k}}\Delta b_{k}+J_{\gamma_{k}}\Delta \gamma_{k}+\epsilon .$$

Assuming that the weighting matrix is $P=\Sigma^{-1}$, the generalized least-squares objective function corresponding to (18) is given by

(19) $$Q_{k}\left(\Delta b_{k},\Delta \gamma_{k}\right)=\Phi_{k}^{T}P\Phi_{k},$$

where $\Phi_{k}=\left(l_{k}-J_{b_{k}}\Delta b_{k}-J_{\gamma_{k}}\Delta \gamma_{k}\right)$. Differentiating (19) with respect to $\Delta b_{k}$ and $\Delta \gamma_{k}$ and setting both derivatives to zero yields the joint normal equations

(20) $$\left[\begin{array}{cc} J_{b_{k}}^{T}PJ_{b_{k}} & J_{b_{k}}^{T}PJ_{\gamma_{k}}\\ J_{\gamma_{k}}^{T}PJ_{b_{k}} & J_{\gamma_{k}}^{T}PJ_{\gamma_{k}} \end{array}\right]\left[\begin{array}{c} \Delta {\hat{b}}_{k}\\ \Delta {\hat{\gamma }}_{k} \end{array}\right]=\left[\begin{array}{c} J_{b_{k}}^{T}Pl_{k}\\ J_{\gamma_{k}}^{T}Pl_{k} \end{array}\right].$$

Equation (20) shows that the spline coefficients and systematic-error parameters are jointly estimated within the same normal-equation system. In the linearized model (16), the parameter perturbations $\Delta b_{k}$ and $\Delta \gamma_{k}$ affect the observations through the sensitivity matrices $J_{b_{k}}$ and $J_{\gamma_{k}}$, respectively. Independent identification of the systematic-error parameters therefore depends on the separability of the column spaces of $J_{b_{k}}$ and $J_{\gamma_{k}}$. This condition is formalized in Theorem 1.

**Theorem** **1.** *If there exist non-zero vectors $d\in R^{3q}$ and $a\in R^{n_{\gamma }}$ such that*

(21) $$J_{\gamma_{k}}a=J_{b_{k}}d,$$

*then the parameter-correction pair $\left(b_{k},\gamma_{k}\right)$ is not locally identifiable from the linearized observation model.*

**Proof of Theorem** **1.** For any candidate correction pair $\left(\Delta b_{k},\Delta \gamma_{k}\right)$, the fitted linearized observation response is $J_{b_{k}}\Delta b_{k}+J_{\gamma_{k}}\Delta \gamma_{k}$. Define another parameter correction pair as

(22) $$\Delta b_{k}^{\prime }=\Delta b_{k}+d,\;\Delta \gamma_{k}^{\prime }=\Delta \gamma_{k}-a.$$

Then, we have

(23) $$\begin{array}{cc} \; & J_{b_{k}}\Delta b_{k}^{\prime }+J_{\gamma_{k}}\Delta \gamma_{k}^{\prime }\\ \; & =J_{b_{k}}\left(\Delta b_{k}+d\right)+J_{\gamma_{k}}\left(\Delta \gamma_{k}-a\right)\\ \; & =J_{b_{k}}\Delta b_{k}+J_{\gamma_{k}}\Delta \gamma_{k}+J_{b_{k}}d-J_{\gamma_{k}}a. \end{array}$$

From (21), we have $J_{b_{k}}d-J_{\gamma_{k}}a=0$. Thus, (23) reduces to

(24) $$J_{b_{k}}\Delta b_{k}^{\prime }+J_{\gamma_{k}}\Delta \gamma_{k}^{\prime }=J_{b_{k}}\Delta b_{k}+J_{\gamma_{k}}\Delta \gamma_{k}.$$

Substituting (24) into (19), we obtain

(25) $$Q_{k}\left(\Delta b_{k}^{\prime },\Delta \gamma_{k}^{\prime }\right)=Q_{k}\left(\Delta b_{k},\Delta \gamma_{k}\right).$$

Because d≠0 and a≠0, we have $\left(\Delta b_{k}^{\prime },\Delta \gamma_{k}^{\prime }\right)\neq \left(\Delta b_{k},\Delta \gamma_{k}\right)$. Therefore, two different parameter correction pairs yield identical fitted values and identical objective function values. Hence, the parameter decomposition is not unique, i.e., the systematic-error parameters are not uniquely identifiable in the generalized least-squares sense. This completes the proof.    □

Theorem 1 indicates that when $R\left(J_{b_{k}}\right)\cap R\left(J_{\gamma_{k}}\right)\neq \left\{0\right\}$, the observation responses induced by spline-coefficient and systematic-error perturbations are indistinguishable along the shared subspace, where R(·) denotes the column space of a matrix.

In practice, exact equality in (21) rarely holds, whereas an approximate relationship, $J_{\gamma_{k}}a\approx J_{b_{k}}d$, is more common. In this case, the systematic-error parameters may remain formally identifiable, but the near-collinearity of the two response spaces makes the normal equations severely ill-conditioned. Consequently, the parameter estimates become highly sensitive to measurement noise, knot placement, and initial values, indicating weak identifiability of the systematic-error parameters.

To characterize the component of the systematic-error response that can be absorbed by spline-coefficient perturbations, define the following weighted projection onto $R(J_{b_{k}})$.

**Definition** **1.** *Let P be symmetric positive definite and define the weighted inner product by ${\langle u,v\rangle }_{P}=u^{T}Pv$. If $J_{b_{k}}$ has full column rank, then $J_{b_{k}}^{T}PJ_{b_{k}}$ is invertible. The matrix*

(26) $$\Pi_{b_{k}}=J_{b_{k}}{\left(J_{b_{k}}^{T}PJ_{b_{k}}\right)}^{-1}J_{b_{k}}^{T}P$$

*is the P-weighted orthogonal projector onto the spline sensitivity space $R(J_{b_{k}})$. The corresponding complementary projection matrix is defined as*

(27) $$M_{b_{k}}=I-\Pi_{b_{k}}.$$

*The range of $M_{b_{k}}$ is the P-weighted orthogonal complement of the spline sensitivity space, namely*

(28) $$R{\left(J_{b_{k}}\right)}^{{⊥}_{P}}=\left\{v:J_{b_{k}}^{T}Pv=0\right\}.$$

Based on Definition 1, the systematic-error response can be decomposed relative to the spline sensitivity space as follows.

**Theorem** **2.** *Under the linearized model in (16), let the observation response induced by the systematic-error parameter perturbation $\Delta \gamma_{k}$ be denoted by $s_{k}=J_{\gamma_{k}}\Delta \gamma_{k}$. Then, the systematic-error response can be decomposed as*

(29) $$s_{k}=\Pi_{b_{k}}s_{k}+M_{b_{k}}s_{k}.$$

*Furthermore, there exists a vector $c_{k}$ such that $\Pi_{b_{k}}J_{\gamma_{k}}\Delta \gamma_{k}=J_{b_{k}}c_{k}$. Accordingly, the linearized observation equation can be reformulated as*

(30) $$l_{k}=J_{b_{k}}\Delta {\tilde{b}}_{k}+M_{b_{k}}J_{\gamma_{k}}\Delta \gamma_{k}+\epsilon ,$$

*where $\Delta {\tilde{b}}_{k}=\Delta b_{k}+c_{k}$.*

**Proof of Theorem** **2.** From Definition 1, $\Pi_{b_{k}}$ is the P-weighted projection matrix onto the spline sensitivity space $R(J_{b_{k}})$, and $M_{b_{k}}=I-\Pi_{b_{k}}$. Therefore, the systematic-error response $s_{k}$ can be decomposed into its projected component in the spline sensitivity space and its complementary component, which gives (29).

By the definition of $\Pi_{b_{k}}$, the projected component of the systematic-error response can be written as

(31) $$\begin{array}{cc} \Pi_{b_{k}}J_{\gamma_{k}}\Delta \gamma_{k} & =J_{b_{k}}{\left(J_{b_{k}}^{T}PJ_{b_{k}}\right)}^{-1}J_{b_{k}}^{T}PJ_{\gamma_{k}}\Delta \gamma_{k}. \end{array}$$

Let $c_{k}={\left(J_{b_{k}}^{T}PJ_{b_{k}}\right)}^{-1}J_{b_{k}}^{T}PJ_{\gamma_{k}}\Delta \gamma_{k}$. Thus, the projected component can be expressed as $\Pi_{b_{k}}J_{\gamma_{k}}\Delta \gamma_{k}=J_{b_{k}}c_{k}$. Therefore, the projected component can be reproduced by the spline-coefficient perturbation $c_{k}$ through the response $J_{b_{k}}$.

Substituting (29) into the linearized observation equation $l_{k}=J_{b_{k}}\Delta b_{k}+J_{\gamma_{k}}\Delta \gamma_{k}+\epsilon$ yields

(32) $$\begin{array}{cc} l_{k} & =J_{b_{k}}\Delta b_{k}+J_{b_{k}}c_{k}+M_{b_{k}}J_{\gamma_{k}}\Delta \gamma_{k}+\epsilon \\ \; & =J_{b_{k}}\left(\Delta b_{k}+c_{k}\right)+M_{b_{k}}J_{\gamma_{k}}\Delta \gamma_{k}+\epsilon . \end{array}$$

By defining $\Delta {\tilde{b}}_{k}=\Delta b_{k}+c_{k}$, (30) is obtained. This completes the proof.    □

Theorem 2 indicates that the component of the systematic-error response lying in the spline sensitivity space $R(J_{b_{k}})$ can be expressed as $J_{b_{k}}c_{k}$ and absorbed into the modified spline-coefficient correction $\Delta {\tilde{b}}_{k}=\Delta b_{k}+c_{k}$. Therefore, the apparent absorption of systematic errors by spline coefficients arises from projecting systematic-error responses onto the spline sensitivity space. The projected component is indistinguishable from a spline-coefficient correction, whereas $M_{b_{k}}J_{\gamma_{k}}\Delta \gamma_{k}$ represents the component that cannot be explained by spline-coefficient perturbations and therefore contains the independent information available for estimating the systematic-error parameters.

**Figure 2:**
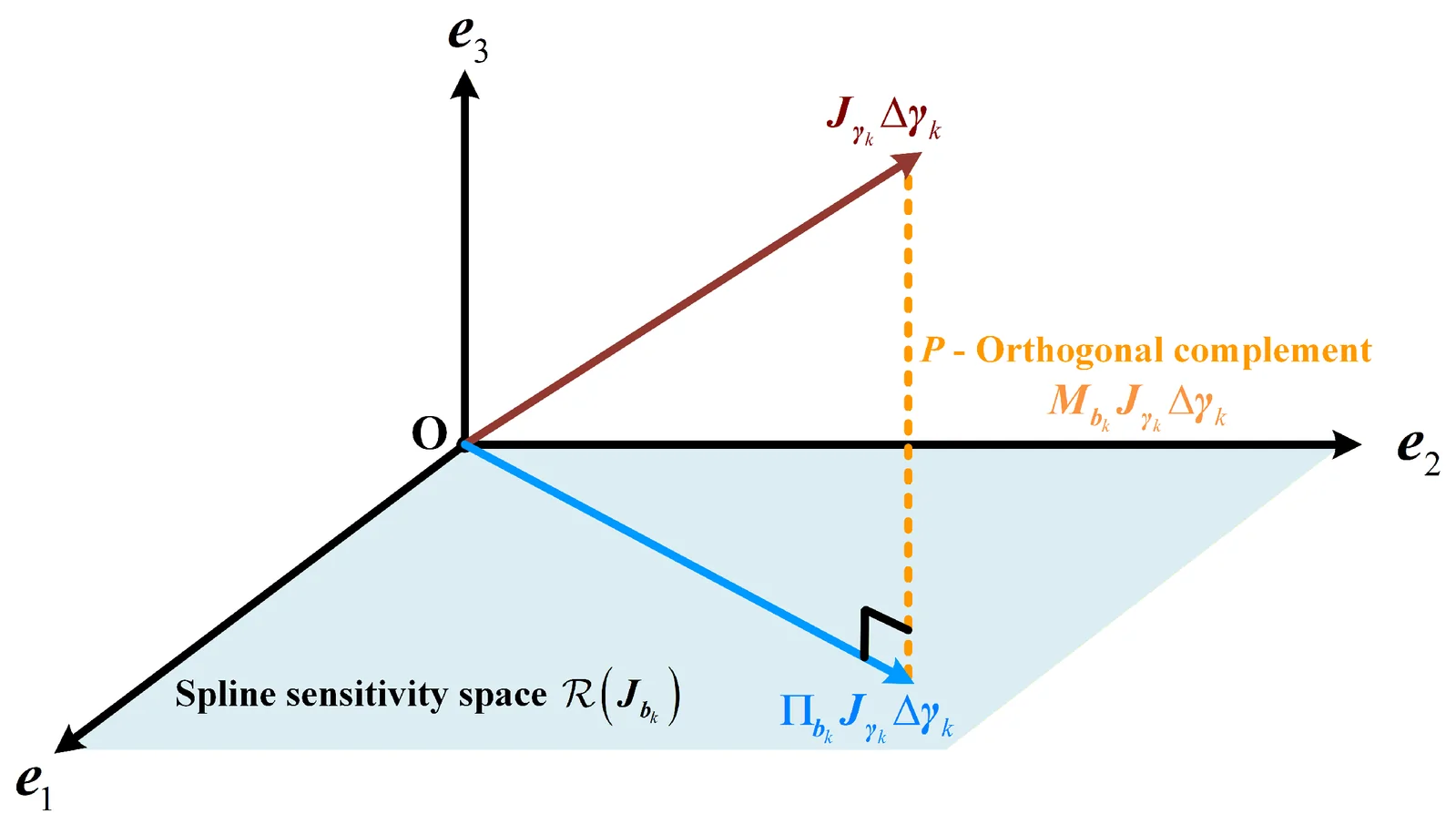
Geometric decomposition of the systematic error response in the observation space Y.

Figure 2 illustrates the geometric decomposition of the systematic-error response $J_{\gamma_{k}}\Delta \gamma_{k}$ in the observation space.

Eliminating the spline-coefficient corrections from the objective function yields a profiled formulation for estimating the systematic-error parameters. For a fixed $\Delta \gamma_{k}$, define $r_{\gamma ,k}=l_{k}-J_{\gamma_{k}}\Delta \gamma_{k}$. Differentiating (19) with respect to $\Delta b_{k}$ gives

(33) $$\frac{\partial Q_{k}}{\partial \Delta b_{k}}=-2J_{b_{k}}^{T}P\left(l_{k}-J_{b_{k}}\Delta b_{k}-J_{\gamma_{k}}\Delta \gamma_{k}\right).$$

Setting this derivative to zero yields the optimal spline-coefficient correction for the given $\Delta \gamma_{k}$

(34) $$\Delta {\hat{b}}_{k}\left(\Delta \gamma_{k}\right)={\left(J_{b_{k}}^{T}PJ_{b_{k}}\right)}^{-1}J_{b_{k}}^{T}P\left(l_{k}-J_{\gamma_{k}}\Delta \gamma_{k}\right).$$

Substituting this expression into (19) yields the profiled objective function for the systematic-error parameters

(35) $$Q_{k}\left(\Delta \gamma_{k}\right)={\left(l_{k}-J_{\gamma_{k}}\Delta \gamma_{k}\right)}^{T}PM_{b_{k}}\left(l_{k}-J_{\gamma_{k}}\Delta \gamma_{k}\right).$$

After eliminating the spline-coefficient corrections, the information available for estimating the systematic-error parameters is contained in the component projected onto the P-weighted orthogonal complement $R{\left(J_{b_{k}}\right)}^{{⊥}_{P}}$.

**Theorem** **3.** *Under the linearized model (16), suppose that P is symmetric positive definite, $J_{b_{k}}$ has full column rank, and the profiled Schur-complement information matrix $S_{\gamma_{k}}=J_{\gamma_{k}}^{T}PM_{b_{k}}J_{\gamma_{k}}$ is nonsingular. Then, the generalized least-squares estimate of the systematic-error parameters at iteration k is given by*

(36) $$\Delta {\hat{\gamma }}_{k}={\left(J_{\gamma_{k}}^{T}PM_{b_{k}}J_{\gamma_{k}}\right)}^{-1}J_{\gamma_{k}}^{T}PM_{b_{k}}l_{k}.$$

*The corresponding profiled Schur-complement information matrix is $S_{\gamma_{k}}=J_{\gamma_{k}}^{T}PM_{b_{k}}J_{\gamma_{k}}$.*

**Proof of Theorem** **3.** Taking the partial derivative of (35) with respect to $\Delta \gamma_{k}$ yields

(37) $$\frac{\partial Q_{k}}{\partial \Delta \gamma_{k}}=-2J_{\gamma_{k}}^{T}PM_{b_{k}}\left(l_{k}-J_{\gamma_{k}}\Delta \gamma_{k}\right).$$

Setting this derivative to zero gives the normal equation

(38) $$J_{\gamma_{k}}^{T}PM_{b_{k}}J_{\gamma_{k}}\Delta {\hat{\gamma }}_{k}=J_{\gamma_{k}}^{T}PM_{b_{k}}l_{k}.$$

By the nonsingularity of the profiled Schur-complement information matrix $S_{\gamma_{k}}$, the generalized least-squares estimate of the systematic-error parameters is given by (36), which completes the proof.    □

Theorem 3 shows that the local identifiability of the systematic-error parameters is determined not by $J_{\gamma_{k}}$ alone, but by the projected sensitivity matrix $M_{b_{k}}J_{\gamma_{k}}$, which represents the component of $J_{\gamma_{k}}$ in the P-weighted orthogonal complement $R{\left(J_{b_{k}}\right)}^{{⊥}_{P}}$ of the spline sensitivity space. If $M_{b_{k}}J_{\gamma_{k}}=0$, then $S_{\gamma_{k}}=0$, and the systematic-error parameters are completely unidentifiable. If $M_{b_{k}}J_{\gamma_{k}}\neq 0$, but the profiled Schur-complement information matrix $S_{\gamma_{k}}$ is nearly singular, the systematic-error parameters are formally estimable but only weakly identifiable, and their estimates are highly sensitive to noise.

Under the local linear approximation, the covariance of the systematic-error parameter estimate is approximated by

(39) $$\mathrm{Cov}\left(\Delta {\hat{\gamma }}_{k}\right)\approx {\left(J_{\gamma_{k}}^{T}PM_{b_{k}}J_{\gamma_{k}}\right)}^{-1}=S_{\gamma_{k}}^{-1}.$$

The approximate equality in (39) arises from the first-order linearization of the original nonlinear estimation model. As $S_{\gamma_{k}}$ approaches singularity, the variance of the systematic-error estimates increases rapidly. In this case, even a numerically converged solution may differ substantially from the true parameter values. The component of the systematic-error response lying in the spline sensitivity space is absorbed into the spline-coefficient correction. Therefore, the final trajectory estimate may contain not only the true trajectory variation but also a portion of the systematic error.

The preceding analysis clarifies the mechanism underlying the weak identifiability of the systematic-error parameters in the B-spline-constrained EMBET model. As shown in (18), perturbations in the spline coefficients and systematic-error parameters jointly contribute to the observation residuals. Their separate identification therefore depends on whether the corresponding observation responses can be distinguished in the observation space. Theorem 1 shows that if the column spaces of the spline sensitivity matrix $J_{b_{k}}$ and the systematic-error sensitivity matrix $J_{\gamma_{k}}$ overlap, the two types of parameter perturbations produce indistinguishable responses along the shared subspace, leading to a non-unique decomposition of the linearized observation response. Theorem 2 further shows that the component of the systematic-error response lying in $R(J_{b_{k}})$ can be represented as $J_{b_{k}}c_{k}$ and absorbed into the spline-coefficient correction. Only the component remaining in the P-weighted orthogonal complement of the spline sensitivity space provides independent information for estimating the systematic-error parameters.

Theorem 3 formalizes this result through the profiled Schur-complement information matrix $S_{\gamma_{k}}=J_{\gamma_{k}}^{T}PM_{b_{k}}J_{\gamma_{k}}$. When the systematic-error sensitivity space is largely aligned with the spline sensitivity space, the projected sensitivity matrix $M_{b_{k}}J_{\gamma_{k}}$ becomes small in norm or nearly rank-deficient. Consequently, $S_{\gamma_{k}}$ becomes ill-conditioned, and the variance of the systematic-error parameter estimates increases. Thus, the absorption of systematic-error responses into the spline-coefficient correction is not merely a numerical artifact of the optimization procedure but a structural consequence of the overlap between the spline and systematic-error sensitivity spaces.

The degree of coupling varies across systematic-error types because their observation-domain responses overlap differently with the spline sensitivity space. For the constant biases, linear drifts, and saturating exponential drifts considered here, the severity of weak identifiability depends on the extent to which their observation-domain responses overlap with the spline sensitivity space. In particular, the smooth time-varying response of a saturating exponential drift may be partially represented by perturbations in the B-spline coefficients, thereby weakening the local identifiability of the corresponding systematic-error parameters. The above analysis shows that weak identifiability in the B-spline-constrained EMBET framework arises from the joint estimation of B-spline coefficients and systematic-error parameters. To avoid this coupling, the proposed method does not estimate systematic errors through the EMBET parameter-estimation process; instead, ResCompFormer directly predicts the systematic-error sequence from observation residuals, thereby avoiding the weak-identifiability mechanism associated with the joint parametric estimation of trajectory and systematic-error terms.

## 3. ResCompFormer-Based Iterative Compensation Method for Systematic Errors

Section 2 established a B-spline-constrained EMBET model for trajectory estimation. Conventional EMBET methods fuse multi-sensor observations by jointly estimating the B-spline coefficients and systematic-error parameters, thereby improving the continuity and stability of trajectory reconstruction. However, when the systematic-error sensitivity space overlaps with the spline sensitivity space, the two parameter groups may produce similar observation responses, resulting in parameter coupling during joint estimation. Consequently, part of the systematic-error response may be absorbed into the spline-coefficient correction, thereby weakening the local identifiability of the systematic-error parameters. To address this issue, the proposed method no longer jointly estimates the B-spline coefficients and systematic-error parameters within the same nonlinear least-squares problem. Instead, ResCompFormer, parameterized by the optimized network parameter set $\theta^{*}$, predicts the signed observation-domain systematic-error sequence from the observation residual sequence while $\theta^{*}$ is held fixed during inference. The B-spline coefficients are then re-estimated from the corrected observations. Consequently, the systematic-error estimate and the B-spline coefficients no longer compete to explain the same observation residuals within a single normal-equation system. This decouples systematic-error estimation from B-spline trajectory estimation and thereby avoids the weak-identifiability mechanism associated with their joint parametric estimation. Within this decoupled formulation, ResCompFormer exploits temporal patterns of systematic errors, sensor identities, measurement-type information, and cross-channel residual structures learned from the training data to predict the observation-domain systematic-error sequence. These learned regularities provide a data-driven prior that improves prediction stability over the data distribution represented during training.

### 3.1. Algorithmic Procedure

The proposed method addresses the coupling between systematic-error parameters and B-spline coefficients through iterative compensation in the observation domain. At each iteration, the residual sequence is fed into the trained ResCompFormer network, which predicts the signed systematic-error sequence under the current trajectory estimate. Let θ denote the set of all trainable parameters of ResCompFormer, and let $\theta^{*}$ denote the optimized parameter set obtained by minimizing the total training loss, as formally defined in Equation (79). During inference, $\theta^{*}$ is held fixed, and the predicted systematic-error sequence is not introduced as an additional unknown to be jointly optimized with the B-spline coefficients. Only the B-spline coefficients are re-estimated by the EMBET solver after the observations have been compensated using the predicted systematic-error sequence. This separates network-based systematic-error estimation from spline-based trajectory estimation at the optimization level.

The stacked observation residual vector at iteration *k* is defined as

(40) $$R_{k}=Y-F\left(b_{k}\right),\;R_{k}\in R^{mC},$$

where $F(b_{k})$ denotes the stacked model-predicted observation vector computed from the current B-spline coefficients $b_{k}$.

For observation channel (i,d), where $d\in D_{i}$, the residual at time *t* is given by

(41) $$r_{i,d}^{\left(k\right)}\left(t\right)=y_{i,d}\left(t\right)-f_{i,d}\left(b_{k},t\right),$$

where $y_{i,d}\left(t\right)$ denotes the measured value of component *d* provided by sensor *i* at time *t* and $f_{i,d}\left(b_{k},t\right)$ denotes the corresponding model-predicted measurement computed from the current B-spline coefficients $b_{k}$.

The initial B-spline coefficients are estimated from the original observations while ignoring systematic errors:

(42) $$b_{0}=\underset{b}{arg min}{∥Y-F\left(b\right)∥}_{P}^{2}.$$

The initial systematic-error estimate is set to ${\hat{U}}_{-1}=0$. Then, for iteration k=0,1,…, the observation residual vector $R_{k}$ is constructed according to (40). According to the fixed observation-channel ordering, the residuals are normalized by their corresponding measurement-noise standard deviations and organized into the matrix

(43) $${\bar{R}}_{k}={\left[{\bar{r}}_{c}^{\left(k\right)}\left(t_{j}\right)\right]}_{\begin{array}{c} j=1,\ldots ,m\\ c=1,\ldots ,C \end{array}}\in R^{m\times C},$$

where

(44) $${\bar{r}}_{c}^{\left(k\right)}\left(t_{j}\right)=\frac{r_{c}^{\left(k\right)}\left(t_{j}\right)}{\sigma_{c}},$$

and observation channel *c* corresponds to a sensor-component pair $\left(i_{c},d_{c}\right)$ under the fixed channel ordering, with $\sigma_{c}\equiv \sigma_{i_{c},d_{c}}$ denoting the corresponding measurement-noise standard deviation.

In the simulation setting considered here, random-noise samples are generated independently across sensors, measurement components, and observation epochs. The corresponding covariance matrix Σ is therefore diagonal, and channel-wise division by $\sigma_{c}$ provides a noise-scale standardization consistent with this covariance structure. This standardization does not imply that the residuals are statistically independent, because systematic errors and trajectory-estimation errors may still introduce temporal and cross-channel correlations.

The trained ResCompFormer maps the standardized residual matrix ${\bar{R}}_{k}$ to a signed observation-domain systematic-error matrix over all sampling epochs and available channels. Although the input residuals are normalized by their measurement-noise standard deviations, the network output remains in the original observation units and can therefore be used directly for observation compensation. The forward prediction is written as

(45) $${\tilde{U}}_{k,\mathrm{mat}}=T_{\theta^{*}}\left({\bar{R}}_{k}\right)\in R^{m\times C},$$

where $T_{\theta^{*}}\left(\cdot \right)$ denotes the trained ResCompFormer network with fixed parameters $\theta^{*}$, *m* is the number of sampling epochs, and *C* is the number of available observation channels. Each row of ${\tilde{U}}_{k,\mathrm{mat}}$ represents the predicted signed systematic-error values corresponding to one sampling epoch, while each column represents the predicted signed systematic-error sequence associated with one available observation channel.

Because the subsequent EMBET trajectory re-estimation uses the vectorized global observation Y, the predicted systematic-error matrix is reshaped into a vector using the same observation ordering:

(46) $${\tilde{U}}_{k}=\mathrm{vec}\left({\tilde{U}}_{k,\mathrm{mat}}\right)\in R^{mC}.$$

To improve iterative stability, a damping update is applied:

(47) $${\hat{U}}_{k}=\eta {\tilde{U}}_{k}+\left(1-\eta \right){\hat{U}}_{k-1},$$

where η∈(0,1] is the damping factor and ${\hat{U}}_{k}\in R^{mC}$ denotes the vectorized signed systematic-error estimate at iteration *k*.

The original observations are then compensated as

(48) $$Y_{c}^{\left(k\right)}=Y-{\hat{U}}_{k},$$

Here, ${\hat{U}}_{k}$ is the estimated signed additive systematic error in the original observations; subtracting it yields the corrected observation vector $Y_{c}^{\left(k\right)}$.

Based on the compensated observations, the B-spline coefficients are re-estimated as

(49) $$b_{k+1}=\underset{b}{arg min}{∥Y_{c}^{\left(k\right)}-F\left(b\right)∥}_{P}^{2}.$$

The procedure is repeated until the relative changes in both the B-spline coefficients and the systematic-error estimate satisfy the convergence criteria

(50) $$\delta_{b_{k}}=\frac{∥b_{k+1}-b_{k}∥}{\max(∥b_{k}∥,\varepsilon )}<\tau_{b},$$

(51) $$\delta_{U_{k}}=\frac{∥{\hat{U}}_{k}-{\hat{U}}_{k-1}∥}{\max(∥{\hat{U}}_{k-1}∥,\varepsilon )}<\tau_{U},$$

where ε is a small positive constant introduced to avoid division by zero and $\tau_{b}$ and $\tau_{U}$ are the convergence thresholds for the B-spline coefficients and the systematic-error estimate, respectively.

Upon convergence, the algorithm returns the B-spline coefficients $\hat{b}$ and the vectorized signed systematic-error estimate $\hat{U}$. The final trajectory $\hat{X}$ is reconstructed at $t_{1},t_{2},\ldots ,t_{m}$ using the prescribed knot vector, spline order, and estimated B-spline coefficients.

Algorithm 1 summarizes the residual-driven iterative compensation procedure. At each iteration, the algorithm constructs observation residuals from the current trajectory estimate, uses ResCompFormer to predict the signed observation-domain systematic-error sequence, and refines the B-spline coefficients through observation compensation and trajectory re-estimation until convergence.

As shown in Algorithm 1, ResCompFormer is the core component of the iterative compensation framework. At each iteration, it predicts the signed observation-domain systematic error from the normalized residuals according to (45), and this prediction directly influences both observation compensation and trajectory reconstruction. Section 3.2 describes the network architecture in detail.
**Algorithm 1:** Iterative Systematic Error Compensation Based on ResCompFormer
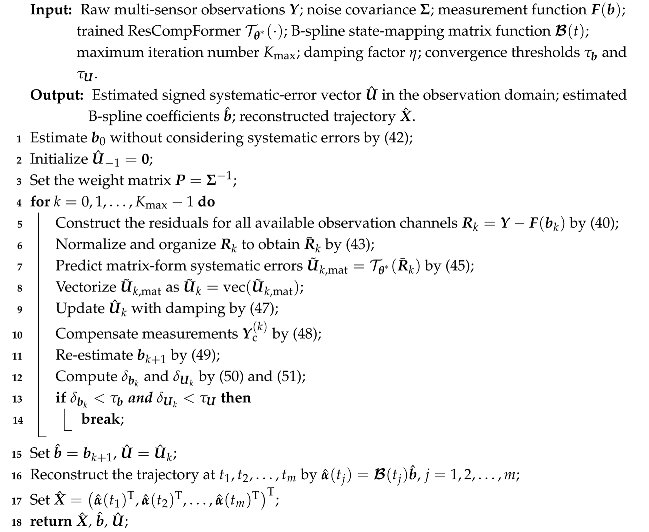


### 3.2. ResCompFormer Network Architecture

ResCompFormer builds on the Transformer architecture to extract features from multi-sensor residual sequences and predict systematic errors. The model comprises a residual-embedding module, an encoder, and a non-autoregressive decoder.

**Figure 3:**
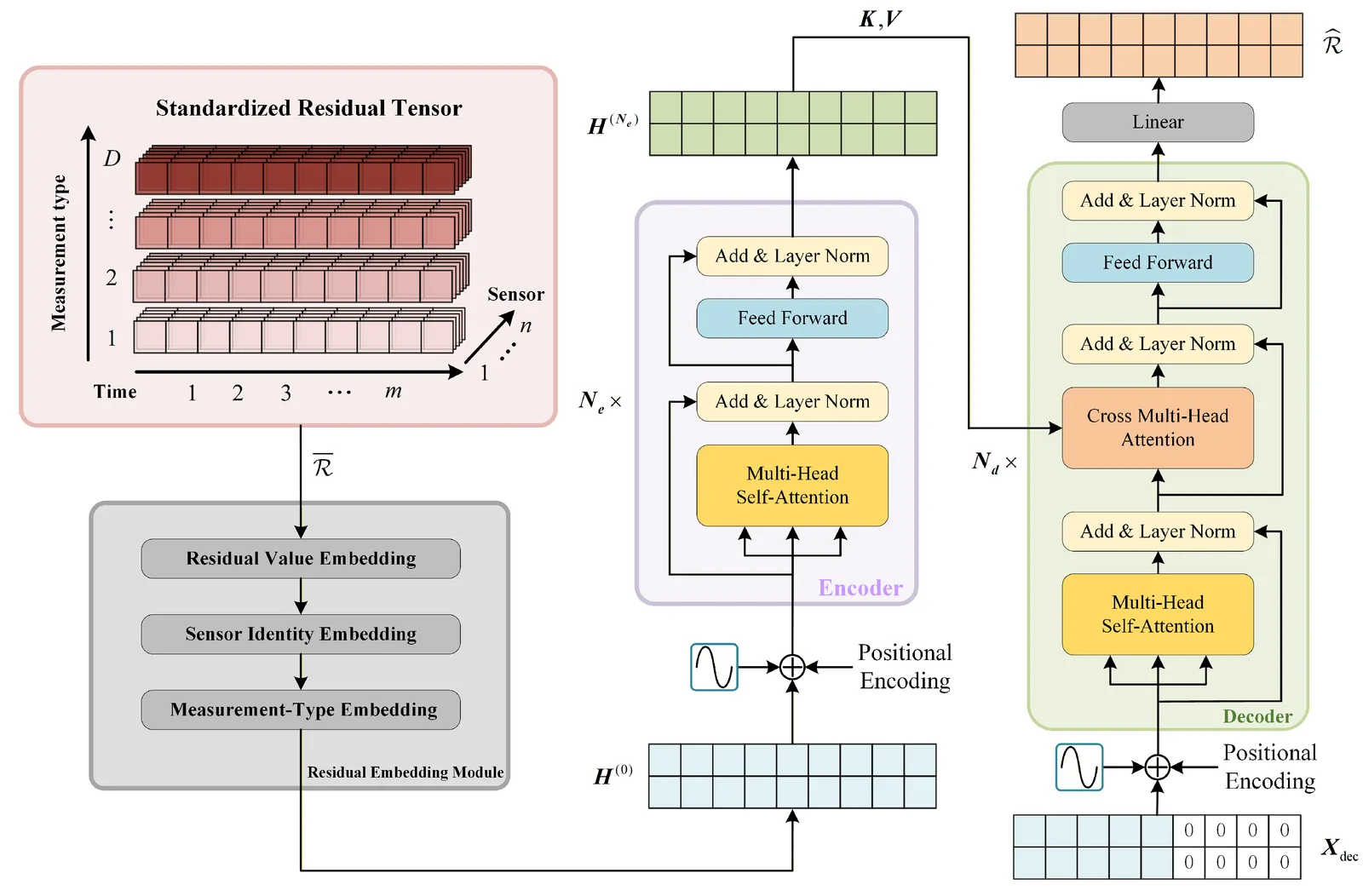
Schematic diagram of the ResCompFormer network architecture.

The residual-embedding module combines sensor identity, measurement type, and numerical residual values in a unified representation. The encoder models temporal dependencies in the fused residual sequence, while the decoder predicts the complete signed systematic-error sequence in parallel within a single forward pass. The overall architecture of ResCompFormer is illustrated in Figure 3.

#### 3.2.1. Residual Embedding Representation

At each iteration, the multi-sensor residuals are first organized into a unified input representation. Let the set of all available observation channels be denoted by $\Omega =\{\left(i,d\right)|i=1,2,\ldots ,n,\;d\in D_{i}\}$, and let the number of channels be $C=\left|\Omega \right|=\sum_{i=1}^{n}\left|D_{i}\right|$.

The normalized residual matrix ${\bar{R}}_{k}\in R^{m\times C}$ defined in Equation (43) is used as the input to ResCompFormer. At sampling epoch $t_{j}$, its *j*th row collects the normalized residuals of all *C* available observation channels:

(52) $${\bar{R}}_{k}\left[j,:\right]=\left[{\bar{r}}_{1}^{\left(k\right)}\left(t_{j}\right),{\bar{r}}_{2}^{\left(k\right)}\left(t_{j}\right),\ldots ,{\bar{r}}_{C}^{\left(k\right)}\left(t_{j}\right)\right]\in R^{1\times C}.$$

Under the fixed channel ordering, observation channel *c* corresponds to the specific sensor-component pair $(i_{c},d_{c})\in \Omega$.

In addition to the numerical residual embedding, the model uses independent learnable embeddings for sensor identity and measurement type. These embeddings allow the temporal model to distinguish residuals from different sensors and measurement modalities.

First, the normalized residual ${\bar{r}}_{c}^{\left(k\right)}\left(t_{j}\right)$ for observation channel *c* is mapped into a $d_{e}$-dimensional feature space

(53) $$e_{c,j}^{r}=W_{r}{\bar{r}}_{c}^{\left(k\right)}\left(t_{j}\right)+b_{r}+e_{d_{c}}^{\mathrm{meas}}+e_{i_{c}}^{\mathrm{sens}},$$

where $W_{r}\in R^{d_{e}\times 1}$ and $b_{r}\in R^{d_{e}}$ denote the weight matrix and bias vector of the numerical residual embedding, respectively. $e_{d_{c}}^{\mathrm{meas}}\in R^{d_{e}}$ and $e_{i_{c}}^{\mathrm{sens}}\in R^{d_{e}}$ are the learnable embedding vectors corresponding to the measurement type $d_{c}$ and sensor index $i_{c}$ for observation channel *c*, respectively.

At each sampling epoch $t_{j}$, the residual embeddings of all *C* available observation channels are arranged in the fixed channel order to form the residual embedding matrix:

(54) $$E_{j}={\left[e_{1,j}^{r},e_{2,j}^{r},\ldots ,e_{C,j}^{r}\right]}^{T}\in R^{C\times d_{e}}.$$

The matrix $E_{j}$ is flattened into a feature vector and then projected to the encoder dimension. The flattened residual embedding vector is denoted by

(55) $$g_{j}=\mathrm{Flatten}\left(E_{j}\right)\in R^{Cd_{e}},$$

where Flatten(·) denotes the channel-wise concatenation of the residual embedding vectors according to the fixed ordering of the available observation channels. Then, a linear mapping is applied to obtain the input feature at time step *j*

(56) $$z_{j}=W_{f}g_{j}+b_{f}\in R^{d_{\mathrm{model}}},$$

where $W_{f}\in R^{d_{\mathrm{model}}\times \left(Cd_{e}\right)}$ and $b_{f}\in R^{d_{\mathrm{model}}}$ are learnable parameters, and $d_{\mathrm{model}}$ denotes the input dimension of the encoder.

The flattening and linear projection jointly fuse the observation channels rather than processing them independently. At each sampling epoch, $g_{j}$ contains the residual embeddings of all *C* channels. Equivalently, if $W_{f}$ is partitioned as $W_{f}=\left[W_{f,1},\ldots ,W_{f,C}\right]$, where $W_{f,c}\in R^{d_{\mathrm{model}}\times d_{e}}$, then $z_{j}=\sum_{c=1}^{C}W_{f,c}e_{c,j}^{r}+b_{f}$. Thus, each latent feature can depend on the residual, sensor identity, and measurement type of every available channel at the same sampling epoch. The Transformer encoder subsequently models the temporal evolution of these jointly fused multichannel features.

This mechanism provides implicit cross-channel feature mixing through the all-channel projection rather than an explicit pairwise channel-attention operation. Consequently, the architecture retains a compact temporal-token representation while preserving information from all observation channels before temporal self-attention. Because the baseline dataset also contains a fixed channel–error assignment, the sensor-identity embeddings may encode a persistent statistical association between particular channel identities and error types. The effect of this association is examined under randomized channel–error assignments in Section 4.4.3.

Finally, positional encoding is added to the input feature $z_{j}$ at each time step to preserve temporal sequence information

(57) $$h_{j}=\mathrm{Dropout}\left(z_{j}+e_{j}^{\mathrm{pos}}\right),$$

where $e_{j}^{\mathrm{pos}}\in R^{d_{\mathrm{model}}}$ is the learnable positional encoding vector for time step *j*, and Dropout is used to regularize training. The features of all time steps are then assembled into the encoder input matrix

(58) $$H^{\left(0\right)}={\left[h_{1},h_{2},\ldots ,h_{m}\right]}^{T}\in R^{m\times d_{\mathrm{model}}}.$$

#### 3.2.2. Encoder

The ResCompFormer encoder maps the fused multi-sensor residual embeddings to high-dimensional temporal features that provide global temporal context for the non-autoregressive decoder. The encoder consists of $N_{e}$ stacked identical layers, each containing a multi-head self-attention (MHA) module and a position-wise feed-forward network (FFN). Let the input of the encoder be $H^{\left(0\right)}\in R^{m\times d_{\mathrm{model}}}$. For encoder layer *l*, the input is the output of the preceding layer, denoted by $H^{\left(l-1\right)}$.

In encoder layer *l*, the input features are first mapped into the query, key, and value matrices through head-specific linear transformations

(59) $$\left\{\begin{array}{c} Q_{h}^{\left(l\right)}=H^{\left(l-1\right)}W_{Q,h}^{\left(l\right)},\\ K_{h}^{\left(l\right)}=H^{\left(l-1\right)}W_{K,h}^{\left(l\right)},\\ V_{h}^{\left(l\right)}=H^{\left(l-1\right)}W_{V,h}^{\left(l\right)}, \end{array}\right.$$

where $h=1,2,\ldots ;N_{h}$ denotes the index of the attention head; and $N_{h}$ is the number of attention heads. $W_{Q,h}^{\left(l\right)}$, $W_{K,h}^{\left(l\right)}$, and $W_{V,h}^{\left(l\right)}\in R^{d_{\mathrm{model}}\times d_{k}}$ are learnable projection matrices for attention head *h* in layer *l*.

Each attention head then models dependencies across time steps using scaled dot-product attention

(60) $${\mathrm{head}}_{h}^{\left(l\right)}=\mathrm{Softmax}\left(\frac{Q_{h}^{\left(l\right)}{\left(K_{h}^{\left(l\right)}\right)}^{T}}{\sqrt{d_{k}}}\right)V_{h}^{\left(l\right)},$$

where $d_{k}=d_{\mathrm{model}}/N_{h}$ denotes the feature dimension of each attention head.

The outputs of all attention heads are concatenated and then projected by an output projection matrix to obtain the output of the multi-head self-attention module

(61) $$\mathrm{MHA}\left(H^{\left(l-1\right)}\right)=\mathrm{Concat}\left({\mathrm{head}}_{1}^{\left(l\right)},\ldots ,{\mathrm{head}}_{N_{h}}^{\left(l\right)}\right)W_{O}^{\left(l\right)},$$

where $W_{O}^{\left(l\right)}\in R^{N_{h}d_{k}\times d_{\mathrm{model}}}$ is the output projection matrix in layer *l*.

Through the multi-head attention mechanism, the model captures long-range temporal dependencies in the fused residual sequence. The sensor-identity and measurement-type information has already been incorporated into the residual embeddings and channel-wise feature fusion, enabling the encoder to represent residual patterns associated with different sensors and measurement types. This representation helps the encoder characterize constant biases, linear drifts, and saturating exponential drifts with distinct temporal structures.

To improve training stability, a residual connection and layer normalization are applied after the multi-head self-attention module

(62) $${\tilde{H}}^{\left(l\right)}=\mathrm{LayerNorm}\left(H^{\left(l-1\right)}+\mathrm{MHA}\left(H^{\left(l-1\right)}\right)\right).$$

The position-wise feed-forward network then applies a nonlinear transformation at each time step

(63) $$\mathrm{FFN}\left({\tilde{H}}^{\left(l\right)}\right)={\mathrm{FC}}_{2}\left(\mathrm{GELU}\left({\mathrm{FC}}_{1}\left({\tilde{H}}^{\left(l\right)}\right)\right)\right),$$

where ${\mathrm{FC}}_{1}$ and ${\mathrm{FC}}_{2}$ denote the first and second fully connected (FC) layers, respectively, and GELU denotes the Gaussian error linear unit activation function.

Finally, a residual connection and layer normalization are applied to the feed-forward output to obtain encoder layer *l*:

(64) $$H^{\left(l\right)}=\mathrm{LayerNorm}\left({\tilde{H}}^{\left(l\right)}+\mathrm{FFN}\left({\tilde{H}}^{\left(l\right)}\right)\right).$$

#### 3.2.3. Decoder

After the encoder extracts global temporal features from the residual sequence, ResCompFormer uses a non-autoregressive decoder to predict the signed systematic-error values for all sampling epochs in parallel. Rather than feeding back previous predictions autoregressively, the decoder concatenates a segment of encoded residual features used as start tokens with zero placeholder vectors at the positions to be predicted. Learnable decoder positional embeddings distinguish these placeholder positions, allowing the complete signed systematic-error sequence to be generated in a single forward pass.

First, a residual feature segment with length $L_{\mathrm{start}}$ is selected from the encoder output sequence $H^{\left(N_{e}\right)}$ as the start token sequence of the decoder

(65) $$X_{\mathrm{start}}=H_{1:L_{\mathrm{start}},:}^{\left(N_{e}\right)}\in R^{L_{\mathrm{start}}\times d_{\mathrm{model}}}.$$

The start-token sequence provides residual context for the decoder. Meanwhile, an all-zero placeholder sequence is constructed as

(66) $$X_{0}=0_{m\times d_{\mathrm{model}}}\in R^{m\times d_{\mathrm{model}}}.$$

The start token sequence and the placeholder sequence are first concatenated as

(67) $$X_{\mathrm{dec}}^{0}=\mathrm{Concat}\left(X_{\mathrm{start}},X_{0}\right)\in R^{\left(m+L_{\mathrm{start}}\right)\times d_{\mathrm{model}}}.$$

Then, a learnable decoder positional embedding matrix is added to the concatenated sequence:

(68) $$X_{\mathrm{dec}}=X_{\mathrm{dec}}^{0}+E_{\mathrm{pos}}^{\mathrm{dec}},\;E_{\mathrm{pos}}^{\mathrm{dec}}\in R^{\left(m+L_{\mathrm{start}}\right)\times d_{\mathrm{model}}}.$$

Although the placeholder vectors themselves are initialized as zeros, the added decoder positional embeddings provide explicit temporal-position information for all prediction slots. Therefore, the decoder can distinguish the prediction slots corresponding to different sampling epochs.

Passing the input through $N_{d}$ stacked decoder layers yields the output feature sequence

(69) $$Z_{\mathrm{dec}}=\mathrm{Decoder}\left(X_{\mathrm{dec}},H^{\left(N_{e}\right)}\right)\in R^{\left(m+L_{\mathrm{start}}\right)\times d_{\mathrm{model}}},$$

where $H^{\left(N_{e}\right)}$ serves as the encoder memory for the decoder.

The first $L_{\mathrm{start}}$ positions correspond to start tokens and serve only as decoder context. Therefore, only the features at the final *m* placeholder positions are retained for systematic-error prediction:

(70) $$Z_{\mathrm{pred}}=Z_{\mathrm{dec}}\left[L_{\mathrm{start}}+1:L_{\mathrm{start}}+m,\;:\right]\in R^{m\times d_{\mathrm{model}}}.$$

A final linear projection maps the prediction features to the *C* observation channels, yielding the signed observation-domain systematic-error matrix

(71) $${\tilde{U}}_{k,\mathrm{mat}}=Z_{\mathrm{pred}}W_{u}+1_{m}b_{u}^{T}\in R^{m\times C},$$

where $W_{u}\in R^{d_{\mathrm{model}}\times C}$ and $b_{u}\in R^{C}$ are the learnable output projection parameters, and $1_{m}$ denotes an *m*-dimensional column vector of ones. Here, column *c* of ${\tilde{U}}_{k,\mathrm{mat}}$ corresponds to the predicted signed systematic-error sequence of observation channel *c* over all sampling epochs.

The predicted matrix is then vectorized using the same channel ordering as the global observation vector Y:

(72) $${\tilde{U}}_{k}=\mathrm{vec}\left({\tilde{U}}_{k,\mathrm{mat}}\right)\in R^{mC}.$$

The non-autoregressive decoder predicts the entire systematic-error sequence in parallel within a single forward pass. This formulation avoids recursive error propagation and reduces the inference latency associated with autoregressive decoding.

#### 3.2.4. Loss Function

ResCompFormer is trained with a joint objective that combines a signed systematic-error prediction loss with a trajectory reconstruction loss. The first term supervises observation-domain error prediction directly, whereas the second links the predicted errors to the downstream trajectory-reconstruction objective.

The signed systematic-error prediction loss directly penalizes deviations between the network output and the ground-truth signed systematic error. Let ${\tilde{U}}_{\mathrm{mat}}$ denote the predicted systematic-error matrix and $U_{\mathrm{mat}}$ the corresponding ground-truth matrix. The loss is defined as

(73) $$L_{e}=\frac{1}{N_{\mathrm{obs}}}\sum_{j=1}^{m}\sum_{c=1}^{C}\frac{{\left[{\tilde{u}}_{c}\left(t_{j}\right)-u_{c}\left(t_{j}\right)\right]}^{2}}{\sigma_{c}^{2}},$$

where $N_{\mathrm{obs}}=mC$ denotes the total number of observation elements, and $\sigma_{c}$ denotes the standard deviation of observation noise for channel *c*.

The trajectory reconstruction loss constrains the final trajectory estimate through a differentiable B-spline-constrained EMBET module, thereby linking observation-domain systematic-error prediction to the downstream trajectory reconstruction objective. Let ${\tilde{U}}_{\mathrm{mat}}\left(\theta \right)$ denote the systematic-error sequence predicted by ResCompFormer, where θ denotes the trainable network parameters. The corresponding vectorized prediction and compensated observation vector are given by

(74) $$\tilde{U}\left(\theta \right)=\mathrm{vec}\left[{\tilde{U}}_{\mathrm{mat}}\left(\theta \right)\right],\;Y_{c}\left(\theta \right)=Y-\tilde{U}\left(\theta \right).$$

Given the compensated observations, the B-spline coefficients are re-estimated by solving the conditional weighted nonlinear least-squares problem

(75) $$\hat{b}\left(\theta \right)=\underset{b}{arg min}\frac{1}{2}{∥Y_{c}\left(\theta \right)-F\left(b\right)∥}_{P}^{2},\;P=\Sigma^{-1}.$$

The reconstructed trajectory is subsequently obtained as

(76) $$\hat{X}\left(\theta \right)=B_{s}\hat{b}\left(\theta \right),$$

where $B_{s}$ is the stacked B-spline state-mapping matrix. During training, the nonlinear iterations executed by the EMBET solver are retained in the computational graph, allowing the trajectory reconstruction loss to propagate through the B-spline coefficient estimation procedure. The corresponding backpropagation and numerical implementation are described in Section 3.2.5.

The trajectory reconstruction loss is defined as

(77) $$L_{t}=\frac{1}{m}\sum_{j=1}^{m}\left[\alpha_{x}\frac{{∥\hat{x}\left(t_{j}\right)-x\left(t_{j}\right)∥}_{2}^{2}}{\sigma_{x}^{2}}+\alpha_{\dot{x}}\frac{{∥\hat{\dot{x}}\left(t_{j}\right)-\dot{x}\left(t_{j}\right)∥}_{2}^{2}}{\sigma_{\dot{x}}^{2}}\right],$$

where $\alpha_{x}+\alpha_{\dot{x}}=1$. The constants $\sigma_{x}$ and $\sigma_{\dot{x}}$ are fixed normalization scales for the position and velocity reconstruction errors, respectively and are computed from the root-mean-square scales of the ground-truth position and velocity sequences in the training set.

The total loss function is defined as

(78) $$L\left(\theta \right)=\lambda_{1}L_{e}\left(\theta \right)+\lambda_{2}L_{t}\left(\theta \right).$$

where $\lambda_{1}$ and $\lambda_{2}$ are the weighting coefficients of the systematic-error prediction loss and trajectory reconstruction loss, respectively.

The optimized network parameters are obtained as

(79) $$\theta^{*}=\underset{\theta }{arg min}L\left(\theta \right).$$

The two loss terms provide complementary forms of supervision. The systematic-error prediction loss directly constrains the signed error sequence in the observation domain, whereas the trajectory reconstruction loss evaluates the effect of the predicted errors on the reconstructed position and velocity. Consequently, the gradient of $L_{t}$ is propagated through observation compensation and the differentiable EMBET trajectory-re-estimation procedure to the ResCompFormer parameters.

#### 3.2.5. Gradient Backpropagation and Numerical Implementation of the Differentiable EMBET Layer

The trajectory reconstruction loss depends on the systematic-error prediction through the nonlinear B-spline trajectory-re-estimation procedure. To propagate trajectory-level supervision to ResCompFormer, the nonlinear iterations executed by the EMBET solver during training are retained in the computational graph and differentiated by backpropagation.

For the compensated observation vector $Y_{c}$, let $b^{\left(s\right)}$ denote the B-spline coefficient vector at the *s*-th nonlinear iteration. Here, *s* indexes the inner Gauss–Newton iterations of the differentiable EMBET solver, whereas *k* denotes the outer systematic-error compensation iteration introduced in Section 3.1. The corresponding observation residual and Jacobian with respect to the B-spline coefficients are

(80) $$r^{\left(s\right)}=Y_{c}-F\left(b^{\left(s\right)}\right),\;J_{b^{\left(s\right)}}={\left.\frac{\partial F(b)}{\partial b}\right|}_{b=b^{\left(s\right)}}.$$

Using the weighting matrix $P=\Sigma^{-1}$ defined in Section 2.1, the Gauss–Newton coefficient increment is obtained from

(81) $$\left(J_{b^{\left(s\right)}}^{T}PJ_{b^{\left(s\right)}}\right)\Delta b^{\left(s\right)}=J_{b^{\left(s\right)}}^{T}Pr^{\left(s\right)},$$

followed by

(82) $$b^{\left(s+1\right)}=b^{\left(s\right)}+\Delta b^{\left(s\right)}.$$

After the prescribed nonlinear iterations, the resulting coefficient vector is denoted by $\hat{b}$, and the reconstructed trajectory is

(83) $$\hat{X}=B_{s}\hat{b}.$$

Gradient propagation through the EMBET solver.

The original observation vector Y is independent of the network parameters, whereas the compensated observations satisfy

(84) $$Y_{c}\left(\theta \right)=Y-\tilde{U}\left(\theta \right).$$

Accordingly, application of the chain rule gives the gradient of the trajectory reconstruction loss with respect to the ResCompFormer parameters as

(85) $$\begin{array}{cc} \nabla_{\theta }L_{t}= & {\left(\frac{\partial \tilde{U}}{\partial \theta }\right)}^{T}{\left(\frac{\partial Y_{c}}{\partial \tilde{U}}\right)}^{T}{\left(\frac{\partial \hat{b}}{\partial Y_{c}}\right)}^{T}B_{s}^{T}\nabla_{\hat{X}}L_{t}. \end{array}$$

Since $\partial Y_{c}/\partial \tilde{U}=-I$, Equation (85) can be written as

(86) $$\nabla_{\theta }L_{t}=-{\left(\frac{\partial \tilde{U}}{\partial \theta }\right)}^{T}{\left(\frac{\partial \hat{b}}{\partial Y_{c}}\right)}^{T}B_{s}^{T}\nabla_{\hat{X}}L_{t}.$$

Equation (86) makes the backpropagation path explicit. The trajectory-level gradient is first mapped from the reconstructed trajectory to the estimated B-spline coefficients, then propagated through the nonlinear trajectory re-estimation procedure to the compensated observations, and finally transferred through the systematic-error prediction to the network parameters.

The term $\partial \hat{b}/\partial Y_{c}$ contains the accumulated dependence of all nonlinear iterations on the compensated observations. From Equation (82), the sensitivity between two successive iterations satisfies

(87) $$\begin{array}{cc} \frac{\partial b^{\left(s+1\right)}}{\partial Y_{c}}= & \left[I+\frac{\partial \Delta b^{\left(s\right)}}{\partial b^{\left(s\right)}}\right]\frac{\partial b^{\left(s\right)}}{\partial Y_{c}}+\frac{\partial \Delta b^{\left(s\right)}}{\partial Y_{c}}. \end{array}$$

The first term in Equation (87) accounts for the indirect influence of the compensated observations through the B-spline coefficients obtained in preceding iterations. The second term accounts for their direct influence on the coefficient increment through the current residual. Moreover, $J_{b^{\left(s\right)}}$ itself depends on $b^{\left(s\right)}$; consequently, coefficient changes generated in earlier iterations affect both the residual and the Jacobian in subsequent iterations. These dependencies are retained in the computational graph and evaluated by automatic differentiation.

The coefficient increment in Equation (81) is obtained by solving a linear system at each nonlinear iteration. Differentiating this equation gives

(88) $$\left(J_{b^{\left(s\right)}}^{T}PJ_{b^{\left(s\right)}}\right)d\Delta b^{\left(s\right)}=d\;\left(J_{b^{\left(s\right)}}^{T}Pr^{\left(s\right)}\right)-d\;\left(J_{b^{\left(s\right)}}^{T}PJ_{b^{\left(s\right)}}\right)\Delta b^{\left(s\right)}.$$

Equation (88) shows that the differential of the B-spline coefficient increment can itself be obtained through a linear-system solution. Therefore, differentiation through the Gauss–Newton step does not require explicitly differentiating a matrix-inverse expression. In practice, the linear-system operation is kept in the computational graph, and its reverse-mode derivatives are evaluated together with the residual, Jacobian, and coefficient-update operations.

Several implementation choices improve the numerical robustness of the differentiable EMBET layer. First, the weighting matrix $P=\Sigma^{-1}$ accounts for the different noise scales of heterogeneous measurement components and reduces scale imbalance in the weighted least-squares system. Second, the coefficient increments and the corresponding backward derivatives are evaluated through linear-system solves rather than through explicitly formed matrix inverses, thereby avoiding unnecessary round-off errors associated with explicit inversion. Third, only the nonlinear iterations executed during training are retained in the computational graph, which limits the depth of the unrolled optimization and reduces the accumulation of numerical and gradient errors over successive iterations.

These measures improve the numerical robustness of the differentiable implementation but do not remove ill-conditioning inherent in the underlying estimation problem. If $J_{b^{\left(s\right)}}$ becomes rank-deficient or nearly rank-deficient, the corresponding linearized least-squares system may remain sensitive to measurement perturbations. The differentiable EMBET layer therefore relies on the same local solvability and conditioning requirements as the underlying nonlinear trajectory estimator.

The differentiable training procedure and the complete inference procedure serve different purposes. During training, the nonlinear trajectory-re-estimation iterations retained in the computational graph provide trajectory-level gradients for network optimization. During inference, the optimized network parameters are held fixed, and the complete residual-driven compensation procedure, including residual construction, systematic-error prediction, observation compensation, and B-spline trajectory re-estimation, is repeated until the prescribed convergence criterion is satisfied.

## 4. Simulation Experiments

This section presents simulation experiments to validate the mechanism underlying weak identifiability analyzed in Section 2.2 and to evaluate the proposed ResCompFormer-based compensation method. After constructing the multi-sensor simulation dataset, a controlled experiment examines the coupling between systematic-error responses and the B-spline trajectory representation. Compensation and trajectory-reconstruction performance are then evaluated under the systematic-error model used to generate the training and test data, with iterative and stepwise EMBET variants included for comparison. The evaluation is further extended to parameter-distribution shift, an unseen systematic-error form, randomized channel–error assignments, and dropout of previously known sensors. Finally, the sensitivity to residual normalization and decoder configuration is examined together with computational efficiency.

### 4.1. Simulation Dataset Construction

#### 4.1.1. UAV Trajectory Construction

To avoid the inverse crime that may arise when the same B-spline representation is used for both trajectory generation and trajectory reconstruction, the ground-truth UAV trajectories are generated using a nonlinear six-degree-of-freedom (six-DOF) fixed-wing flight-dynamics model rather than a prescribed spline model. The flight-dynamics model and representative maneuver scenarios follow established theoretical and simulation formulations for small fixed-wing UAVs [45,46].

For trajectory sample *ℓ*, the full flight-dynamics state used for ground-truth trajectory generation is defined as

(89) $$\chi^{\left(\ell \right)}\left(t\right)={\left[x_{n}^{(\ell )T}\left(t\right),v_{b}^{(\ell )T}\left(t\right),\phi^{\left(\ell \right)}\left(t\right),\theta^{\left(\ell \right)}\left(t\right),\psi^{\left(\ell \right)}\left(t\right),\omega_{x}^{\left(\ell \right)}\left(t\right),\omega_{y}^{\left(\ell \right)}\left(t\right),\omega_{z}^{\left(\ell \right)}\left(t\right)\right]}^{T},$$

where $x_{n}^{\left(\ell \right)}\left(t\right)$ denotes the UAV position in the local north-east-down (NED) navigation frame; $v_{b}^{\left(\ell \right)}\left(t\right)$ denotes the velocity expressed in the body-fixed frame; and $\phi^{\left(\ell \right)}\left(t\right)$, $\theta^{\left(\ell \right)}\left(t\right)$, and $\psi^{\left(\ell \right)}\left(t\right)$ denote the roll, pitch, and yaw angles, respectively. Furthermore, $\omega_{x}^{\left(\ell \right)}\left(t\right)$, $\omega_{y}^{\left(\ell \right)}\left(t\right)$, and $\omega_{z}^{\left(\ell \right)}\left(t\right)$ denote the corresponding body-axis angular rates.

The attitude angles and angular rates in Equation (89) are auxiliary states required by the six-DOF flight-dynamics simulator to generate dynamically feasible trajectories. They are neither included in the heterogeneous multi-sensor observation model nor estimated by the EMBET solver. Only the position and velocity components generated by the flight-dynamics model are retained for observation generation and subsequent trajectory reconstruction.

The state evolution is governed by

(90) $${\dot{\chi }}^{\left(\ell \right)}\left(t\right)=F\left(\chi^{\left(\ell \right)}\left(t\right),\delta^{\left(\ell \right)}\left(t\right);\eta \right),$$

where F(·) represents the nonlinear six-DOF flight-dynamics model; $\delta^{\left(\ell \right)}\left(t\right)$ contains the throttle, aileron, elevator, and rudder commands; and η collects the aircraft mass, moments of inertia, aerodynamic coefficients, propulsion parameters, and gravity-related parameters.

To construct trajectory samples with diverse motion characteristics, the initial position, heading, airspeed, altitude, commanded flight states, maneuver initiation time, and maneuver duration are independently randomized within prescribed feasible ranges. A guidance and stabilization module converts the commanded heading, airspeed, altitude, and turning variables into dynamically feasible throttle and control-surface commands. Accordingly, the resulting dataset covers straight and level flight, climbing and descending flight, coordinated turns, loitering flight, and coupled three-dimensional maneuvers with diverse spatial geometries and motion intensities.

The nonlinear dynamic equations are numerically integrated over the time interval $\left[0,T_{\max}\right]$ using the sampling interval $T_{s}$. Trajectory samples that violate the prescribed airspeed, altitude, or bank-angle constraints are discarded and regenerated.

Because the velocity state in Equation (89) is expressed in the body-fixed frame, it is first transformed into the local NED navigation frame as

(91) $$v_{n}^{\left(\ell \right)}\left(t\right)=C_{b}^{n}\left(\phi^{\left(\ell \right)}\left(t\right),\theta^{\left(\ell \right)}\left(t\right),\psi^{\left(\ell \right)}\left(t\right)\right)v_{b}^{\left(\ell \right)}\left(t\right),$$

where $C_{b}^{n}\left(\cdot \right)$ denotes the direction-cosine matrix from the body-fixed frame to the local NED navigation frame.

Finally, the generated NED position $x_{n}^{\left(\ell \right)}\left(t\right)$ and velocity $v_{n}^{\left(\ell \right)}\left(t\right)$ are transformed into the Earth-centered, Earth-fixed (ECEF) frame using a predefined geodetic reference point. Only the resulting ECEF position $x^{\left(\ell \right)}\left(t\right)$ and velocity ${\dot{x}}^{\left(\ell \right)}\left(t\right)$ constitute the trajectory state used to generate the heterogeneous multi-sensor observations and to evaluate the subsequent EMBET trajectory reconstruction. The attitude angles and angular rates are not passed to the EMBET solver.

#### 4.1.2. Multi-Sensor Observation Data Generation

The simulation uses 11 sensors to track the target: seven radar sensors and four electro-optical sensors. The radar measurement components include range and range rate, while the electro-optical sensors measure azimuth and elevation angles. The locations of all sensor stations are preset in the ECEF frame. For trajectory *ℓ* at sampling time $t_{j}$, multi-sensor observations are generated using the sensor model in (1), the ground-truth ECEF position $x^{\left(\ell \right)}\left(t_{j}\right)$ and velocity ${\dot{x}}^{\left(\ell \right)}\left(t_{j}\right)$, and the sensor-station position $s_{i}$, which defines the origin of the corresponding local ENU frame.

To emulate practical measurements, Gaussian white noise and the systematic-error components described below are added to the simulated observations. The random-noise samples are independently generated across sensors, measurement components, and observation epochs. Accordingly, the measurement-noise covariance matrix Σ used in the simulation experiments is diagonal. The standard deviations of the range and range rate measurement noises are set to $\sigma_{R}=5$ m and $\sigma_{\dot{R}}=0.3$ m/s, respectively, while the standard deviations of the azimuth and elevation measurement noises are both set to $\sigma_{A}=5^{\prime \prime }$ and $\sigma_{E}=5^{\prime \prime }$. The parameter settings of the simulation dataset are given in Table 1.

Three types of systematic error are considered: constant bias, linear drift, and saturating exponential drift. For measurement component *d* of sensor *i*, the systematic error is uniformly expressed as

(92) $$u_{i,d}\left(t_{j}\right)=u_{i,d}^{0}+k_{i,d}\Delta t_{j}+\beta_{i,d}\left[1-\exp\left(-\frac{\Delta t_{j}}{\tau_{i,d}^{c}}\right)\right],$$

where $\Delta t_{j}=t_{j}-t_{1}$ denotes the elapsed time from the first sampling instant, $u_{i,d}^{0}$ denotes the constant-bias coefficient, $k_{i,d}$ denotes the linear-drift coefficient, $\beta_{i,d}$ denotes the asymptotic magnitude of the saturating exponential drift, and $\tau_{i,d}^{c}$ denotes the corresponding time constant.

To represent channel-dependent error characteristics in a heterogeneous multi-sensor system, a unique systematic-error type is assigned to each active observation channel. In the unified systematic-error model, this channel-wise configuration is implemented by setting the coefficients associated with the inactive error types to zero. The resulting error-type configuration is given in Table 2. This configuration defines the baseline systematic-error setting used in the controlled analysis of Section 4.2 and the comparative experiment of Section 4.3. Section 4.4 subsequently varies the parameter distribution, error functional form, channel–error assignment, and sensor availability to examine the robustness of the estimation methods beyond this baseline setting. Parameters associated with different observation channels are assigned independently, even when they have the same numerical value or sampling range. All angular systematic-error parameters are converted into radians before numerical calculation.

### 4.2. Controlled Analysis of Weak Identifiability

A paired controlled experiment with a correctly specified systematic-error model is conducted to examine empirically the mechanism underlying weak identifiability analyzed in Section 2.2. The experiment compares a noise-only reference with a scenario in which the prescribed systematic errors are present and jointly estimated with the B-spline trajectory. The same trajectories, random-noise realizations, and trajectory initializations are used in the paired runs. This design quantifies the additional reconstruction and parameter-estimation difficulty associated with the presence and joint estimation of systematic errors without introducing error–model mismatch. The resulting parameter, systematic-response, and trajectory statistics provide a controlled reference for the compensation experiments in Section 4.3.

#### 4.2.1. Experimental Setup

The experiment uses the 11-sensor network described in Section 4.1, including seven radar sensors that provide range and range-rate measurements and four electro-optical sensors that provide azimuth and elevation measurements. All 22 observation channels are retained. The observation channels with active systematic errors and their fixed true parameter values are listed in Table 2 and Table 3. The standard deviations of the random measurement errors are 5m for range, 0.3m/s for range rate, and $5^{\prime \prime }$ for both azimuth and elevation.

Twenty deterministic three-dimensional trajectories are generated, and 30 independent Gaussian-noise realizations are used for each trajectory, resulting in 600 paired runs. In Scenario 1, the observations contain random noise only and the trajectory is estimated without active systematic-error parameters. In Scenario 2, the prescribed constant, linear, and saturating exponential errors are added simultaneously, and the B-spline coefficients and 16 active systematic-error parameters are estimated jointly. The same trajectory, random-noise realization, and initialization are used in both scenarios.

The position and velocity root mean square errors (RMSE) are calculated as

(93) $${\mathrm{RMSE}}_{x}=\sqrt{\frac{1}{m}\sum_{j=1}^{m}{∥\hat{x}\left(t_{j}\right)-x\left(t_{j}\right)∥}_{2}^{2}},\;{\mathrm{RMSE}}_{\dot{x}}=\sqrt{\frac{1}{m}\sum_{j=1}^{m}{∥\hat{\dot{x}}\left(t_{j}\right)-\dot{x}\left(t_{j}\right)∥}_{2}^{2}}.$$

The heterogeneous systematic-error parameters are summarized using the scale-normalized root mean square error (sNRMSE),

(94) $${\mathrm{sNRMSE}}_{\gamma }=\sqrt{\frac{1}{N_{\gamma }}\sum_{a=1}^{N_{\gamma }}{\left(\frac{{\hat{\gamma }}_{a}-\gamma_{a}^{\mathrm{true}}}{s_{a}^{\mathrm{true}}}\right)}^{2}},\;s_{a}^{\mathrm{true}}=\left|\gamma_{a}^{\mathrm{true}}\right|,$$

where $N_{\gamma }=16$ is the number of active systematic-error parameters in Scenario 2, and all active true parameters are nonzero. The mean absolute normalized errors are calculated using the same parameter scale. The absorption ratio of the actual systematic-error response is defined as

(95) $$\eta_{U}=\frac{{∥\Pi_{b}U^{\mathrm{true}}∥}_{P}^{2}}{{∥U^{\mathrm{true}}∥}_{P}^{2}}\times 100\%,$$

where $\Pi_{b}U^{\mathrm{true}}$ denotes the component of the true systematic-error response that lies in the spline sensitivity space.

#### 4.2.2. Experimental Results and Analysis

As shown in Table 4, relative to the paired noise-only reference, the joint-estimation scenario increases the mean position RMSE by approximately 18.8% and the mean velocity RMSE by about 8.3%. The larger relative degradation in position is consistent with the mechanism underlying weak identifiability analyzed in Section 2.2. Slowly varying systematic-error responses can be partially represented by smooth, low-frequency perturbations of the B-spline trajectory, so the trajectory and systematic-error terms compete to explain part of the same observation residual. This reduced separability primarily affects position reconstruction in the present experiment.

The smaller degradation in velocity is consistent with two properties of the present observation model. First, velocity is directly constrained by multiple range-rate channels, including channels without active systematic errors, which provide additional constraints that limit systematic-error-induced trajectory distortion. Second, the derivative-based velocity reconstruction is less sensitive than position to very low-frequency perturbations of the spline trajectory. Consequently, the effect of the joint-estimation scenario is also visible in velocity, but is weaker than that observed in position.

Overall, the experiment shows that weak identifiability primarily degrades position reconstruction and increases uncertainty in systematic-error estimation. Representative parameter-wise estimation statistics are further reported in Table 5. Its effect on velocity is smaller because range-rate measurements provide direct constraints and differentiation attenuates low-frequency position deviations. This behavior persists even when all observation channels are retained and the estimation model exactly matches the systematic-error generation model. The degradation therefore cannot be attributed to error–model mismatch and is consistent with the overlap between the trajectory and systematic-error sensitivity spaces analyzed in Section 2.2. The parameter uncertainty does not necessarily translate directly into an equally large discrepancy in the reconstructed systematic-error sequence. Over a finite observation interval, different parameter combinations can produce similar slowly varying responses, while the component aligned with the spline sensitivity space may be partially absorbed by the trajectory correction. Parameter recovery, systematic-error-sequence recovery, and trajectory reconstruction therefore describe related but distinct aspects of the estimation problem. Section 4.3 compares these quantities under randomized systematic-error parameters.

### 4.3. Systematic Error Compensation and Trajectory Reconstruction Under Matched Conditions

The controlled analysis in Section 4.2 shows that systematic-error responses can overlap with the B-spline trajectory representation during joint estimation. Building on this result, the following experiment compares systematic-error compensation and trajectory reconstruction under the baseline error model in Equation (92), with the nonzero error parameters randomized according to Table 3. All methods are evaluated using the same test trajectories, noise realizations, and systematic-error realizations. Section 4.4 then examines performance when the parameter distribution, error functional form, channel–error assignment, or sensor availability differs from this baseline setting.

#### 4.3.1. Dataset Settings

The trajectory, sensor configuration, observation channels, and measurement noise settings are identical to those described in Section 4.1. Unlike the weak-identifiability experiment, the nonzero systematic-error parameters are independently sampled for each trajectory from the compensation ranges listed in Table 3. Therefore, different samples contain different amplitudes and temporal patterns of constant-bias, linear-drift, and saturating-exponential errors.

The 1000 generated trajectories are divided at the trajectory level into training, validation, and test sets at a ratio of 80%, 10%, and 10%, respectively. The training and validation sets are used only for training and model selection of the neural-network-based methods M5–M9. All methods are evaluated on the same matched-condition test set using identical test trajectories, systematic-error realizations, and random-noise realizations. The prescribed measurement-noise standard deviations used for residual normalization and loss weighting are fixed according to the sensor settings. Any additional data-dependent normalization statistics are calculated exclusively from the training set and then fixed.

The M9 configuration used in the main comparison follows the settings described in Section 3. The sensitivity experiments in Section 4.5 vary the normalization or decoder configuration while keeping the data partition and the remaining training settings unchanged. For the parameter-shift, error–model-mismatch, and sensor-dropout experiments in Section 4.4, the trained M9 parameters are held fixed. A separately trained model is used only in the Random→Random channel-assignment experiment.

#### 4.3.2. Model Training Settings

For M5–M9, the network input is constructed using the residual initialization procedure in Section 3.1. Specifically, the initial trajectory is estimated without explicitly modeling systematic errors, and the normalized residual sequence defined in Equations (40) and (43) is used as the network input. The corresponding signed systematic-error sequence is used as the supervision target. Because the residual is computed from the estimated trajectory rather than the ground-truth trajectory, ground-truth trajectory information is not used as network input.

The ResCompFormer encoder and decoder contain $N_{e}=4$ and $N_{d}=3$ layers, respectively. The embedding dimension is $d_{\mathrm{model}}=512$, the number of attention heads is $N_{h}=8$, and GELU is used as the activation function. The main M9 configuration uses the noise-standardized residual input in Equation (43) and the parallel decoder defined by Equations (65)–(71). Its start-token length is set to $L_{\mathrm{start}}=200$ sampling epochs, denoted by $L_{0}$ in the sensitivity analysis of Section 4.5.3. The systematic-error prediction loss, trajectory reconstruction loss, and total loss follow Equations (73), (77), and (78), respectively. All neural models are implemented in PyTorch 2.6.0 and trained using AdamW. The batch size is set to 8, the initial learning rate is $5\times 10^{-5}$, and the maximum number of training epochs is 100. A warm-up stage followed by cosine annealing is used for learning-rate adjustment, and the model with the lowest validation loss is retained for testing. The experiments are conducted on a computer equipped with an NVIDIA RTX 4070 GPU, 64 GB of memory, and an Intel Core i9-14900 processor.

During testing, the optimized network parameter set $\theta^{*}$ is held fixed. Systematic-error prediction, observation compensation, and B-spline trajectory re-estimation are performed according to Equations (47)–(49). The iterative procedure is terminated when the convergence criteria in Equations (50) and (51) are satisfied.

#### 4.3.3. Comparative Experiments

The standard cubic B-spline EMBET method [1], adaptive-knot B-spline method [19], Bayesian information criterion (BIC)-based free-knot B-spline method [18], and regularized B-spline method [7] are denoted by M1–M4, respectively. A bidirectional long short-term memory (BiLSTM) network [47], a temporal convolutional network (TCN) [48], a standard Transformer [42], and an iTransformer [38] are adapted to predict systematic-error sequences from the same residual inputs and are denoted by M5–M8, respectively. The proposed ResCompFormer is denoted by M9.

Two additional model-driven variants are constructed from M4 to examine the influence of the estimation schedule. M10 performs iterative compensation: the systematic-error parameters are estimated from the current trajectory, the original observations are compensated using the reconstructed systematic-error sequence, and the B-spline trajectory is re-estimated. The residuals are then reconstructed and the procedure is repeated until the outer convergence criteria in Equations (50) and (51) are satisfied. M11 uses the same iterative framework but estimates the systematic-error terms in two stages. Constant-bias terms are estimated and compensated first, followed by the linear and saturating-exponential terms and a new trajectory update. M10 and M11 use the same parametric error model as M4 and contain no learned parameters.

The model-driven methods M1–M4 do not require data-driven training and are applied independently to every trajectory in the test set. For each test trajectory, these methods jointly estimate the B-spline trajectory coefficients and the corresponding systematic-error parameters from the same noisy observations. The estimated systematic-error parameters are then substituted into Equation (92) to reconstruct the complete systematic-error sequences for evaluation. M10 and M11 use the same systematic-error parameterization, initialization, regularization settings, and observation weights as M4. Their outer stopping conditions are consistent with the convergence criteria used by the iterative compensation procedure. Comparisons among M4, M10, and M11 therefore separate one-shot joint estimation, iterative compensation, and stepwise parameter estimation. The neural-network-based methods M5–M9 use the same training, validation, and test partitions, residual inputs, supervision targets, and iterative trajectory re-estimation procedure. After training and model selection using the training and validation sets, respectively, the fixed models are evaluated on exactly the same test trajectories used for the model-driven methods. Consequently, M1–M11 are evaluated using identical test trajectories, systematic-error realizations, and random-noise realizations whenever the method is applicable, and their systematic-error estimates are compared in the same observation domain.

Although the true systematic-error parameters vary among the test samples, all compared methods are evaluated on a paired, sample-by-sample basis. For test trajectory *ℓ*, the trajectory and systematic-error sequence estimated by each method are compared with the corresponding sample-specific ground truth. The resulting sample-level errors are subsequently summarized over the complete test set. Therefore, the comparison reflects paired estimation errors under identical test conditions rather than differences in the absolute parameter values assigned to individual trajectories.

The position and velocity reconstruction errors are evaluated using ${\mathrm{RMSE}}_{x}$ and ${\mathrm{RMSE}}_{\dot{x}}$, respectively. To evaluate heterogeneous systematic-error sequences with different physical units, the noise-normalized root mean square error (nRMSE) of the systematic-error sequence produced by method *r* for test trajectory *ℓ* is defined as

(96) $${\mathrm{nRMSE}}_{U}^{\left(\ell ,r\right)}={\left[\frac{1}{N_{\mathrm{obs}}}\sum_{j=1}^{m}\sum_{c=1}^{C}\frac{{\left({\hat{u}}_{r,c}^{\left(\ell \right)}\left(t_{j}\right)-u_{c}^{\left(\ell \right)}\left(t_{j}\right)\right)}^{2}}{\sigma_{c}^{2}}\right]}^{1/2},$$

where $N_{\mathrm{obs}}=mC$, $u_{c}^{\left(\ell \right)}\left(t_{j}\right)$ denotes the true signed systematic error of channel *c* at time $t_{j}$, and ${\hat{u}}_{r,c}^{\left(\ell \right)}\left(t_{j}\right)$ is the corresponding final estimate produced by method *r*. The noise standard deviation $\sigma_{c}$ is identical to that used in Equation (73).

Unlike ${\mathrm{sNRMSE}}_{\gamma }$, which evaluates the estimated parametric coefficients, ${\mathrm{nRMSE}}_{U}$ evaluates the complete systematic-error response in the observation domain. It is therefore applicable to both parameterized EMBET methods and neural-network methods that directly predict systematic-error sequences.

In the compensation dataset, several systematic-error parameters are sampled from intervals that include values close to zero. Normalizing the estimation error by the sample-specific true parameter magnitude, as in Equation (94), would therefore become unstable for these samples. For the parametric methods, the parameter error is instead normalized by the width of the sampling range. Let the sampling interval of parameter $\gamma_{a}$ in Table 3 be $\left[l_{a},u_{a}\right]$, and define its range width as $w_{a}=u_{a}-l_{a}$. Let $N_{\mathrm{test}}$ denote the number of test trajectories and $N_{\gamma }$ the number of active systematic-error parameters evaluated in the parameter domain. The range-normalized parameter RMSE is

(97) $${\mathrm{pNRMSE}}_{\gamma }^{\left(r\right)}={\left[\frac{1}{N_{\mathrm{test}}N_{\gamma }}\sum_{\ell =1}^{N_{\mathrm{test}}}\sum_{a=1}^{N_{\gamma }}{\left(\frac{{\hat{\gamma }}_{r,a}^{\left(\ell \right)}-\gamma_{a}^{\left(\ell \right)}}{w_{a}}\right)}^{2}\right]}^{1/2},$$

and the corresponding signed normalized bias and mean absolute normalized parameter error are

(98) $${\mathrm{pBias}}_{\gamma }^{\left(r\right)}=\frac{1}{N_{\mathrm{test}}N_{\gamma }}\sum_{\ell =1}^{N_{\mathrm{test}}}\sum_{a=1}^{N_{\gamma }}\frac{{\hat{\gamma }}_{r,a}^{\left(\ell \right)}-\gamma_{a}^{\left(\ell \right)}}{w_{a}},\;{\mathrm{pMAE}}_{\gamma }^{\left(r\right)}=\frac{1}{N_{\mathrm{test}}N_{\gamma }}\sum_{\ell =1}^{N_{\mathrm{test}}}\sum_{a=1}^{N_{\gamma }}\left|\frac{{\hat{\gamma }}_{r,a}^{\left(\ell \right)}-\gamma_{a}^{\left(\ell \right)}}{w_{a}}\right|.$$

These range-normalized metrics are used for the compensation experiment because its randomized parameter intervals may include values close to zero. They are distinct from ${\mathrm{sNRMSE}}_{\gamma }$ in the controlled weak-identifiability experiment, where the true parameters are fixed and nonzero and normalization by $|\gamma_{a}^{\mathrm{true}}|$ is well defined. The two parameter-domain summaries therefore serve complementary purposes and should not be compared numerically as if they used the same normalization. Because M5–M9 directly predict systematic-error sequences rather than the coefficients of Equation (92), parameter-domain metrics are reported only for the parametric model-driven methods.

Table 6 summarizes the overall comparison of trajectory reconstruction and systematic-error estimation performance under the matched test condition.

Table 7 provides a complementary view in the parameter domain. Relative to M4, M10 reduces the reported ${\mathrm{pNRMSE}}_{\gamma }$ and ${\mathrm{pMAE}}_{\gamma }$ by 9.33% and 9.49%, respectively, whereas M11 achieves larger reductions of 19.40% and 20.44%. The signed ${\mathrm{pBias}}_{\gamma }$ remains close to zero for all model-driven methods and does not decrease monotonically, which is expected because positive and negative parameter errors can partially cancel in a signed average. Together with Table 6, these results show that iterative and stepwise estimation improve explicit parameter recovery, but parameter-domain accuracy should still be distinguished from observation-domain systematic-error recovery and downstream trajectory reconstruction.

This distinction is also consistent with the controlled weak-identifiability experiment in Table 4. Although ${\mathrm{sNRMSE}}_{\gamma }$ and ${\mathrm{pNRMSE}}_{\gamma }$ are based on different normalization schemes and therefore cannot be compared numerically, both experiments show that imperfect recovery of the explicit systematic-error parameters does not translate one-to-one into an equally large error in the reconstructed observation-domain systematic-error sequence. This behavior is expected under weak identifiability because different parameter perturbations can generate similar observation responses, while part of the systematic-error response can be absorbed by the B-spline trajectory representation. Consequently, the practical concern is not the exact recovery of every parametric coefficient but whether the remaining ambiguity degrades systematic-error compensation and trajectory reconstruction.

**Figure 4:**
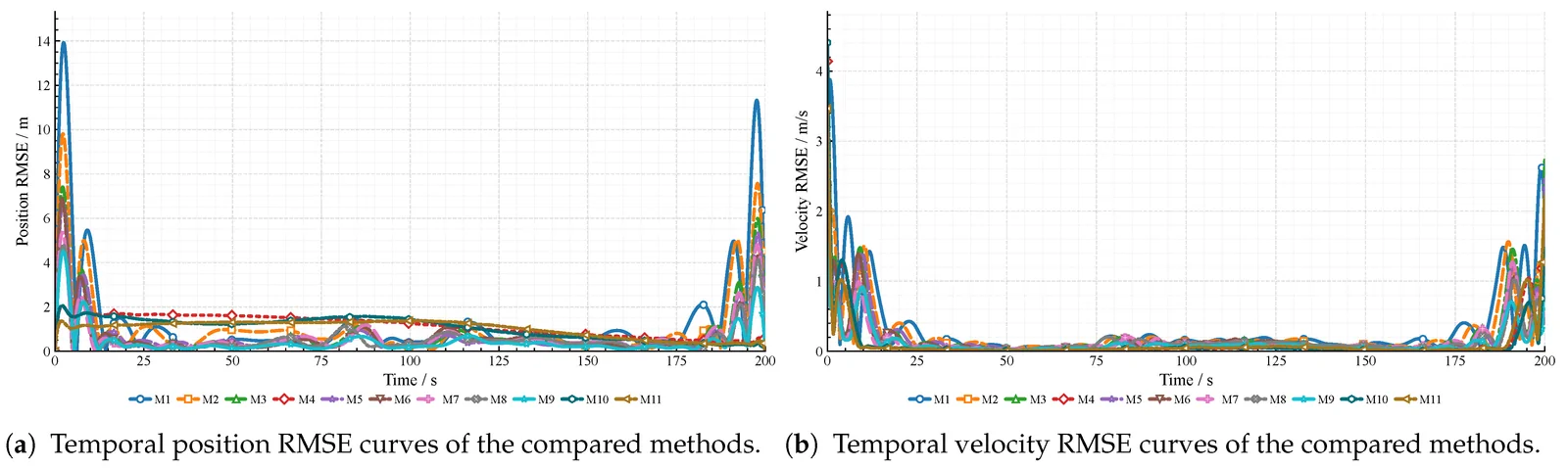
Temporal comparison of trajectory reconstruction errors over the complete observation interval.

The temporal position and velocity RMSE results are further visualized in Figure 4.

As shown in Table 6, under the matched test condition, M9 achieves the lowest ${\mathrm{RMSE}}_{x}$, ${\mathrm{RMSE}}_{\dot{x}}$, and ${\mathrm{nRMSE}}_{U}$ among all compared methods. Compared with the best-performing one-shot model-driven method, M4, M9 reduces the position RMSE by 38.81%. The iterative variants M10 and M11 progressively improve on M4; in particular, M11 reduces the position RMSE from 1.2383 m to 1.0829 m and the systematic-error nRMSE from 5.2811 to 4.7318. Compared with the strongest data-driven baseline, M8, M9 also achieves lower errors across all three metrics. These results establish the matched-condition reference; Section 4.4 examines how the performance changes when the test distribution or sensor configuration departs from this setting.

Figure 4a shows that all methods exhibit relatively large position errors near the beginning and end of the observation interval, whereas the errors remain substantially lower over the central portion of the trajectory. Compared with all other methods, M9 suppresses both the boundary peaks and intermediate fluctuations more effectively, resulting in the lowest overall position RMSE.

A similar temporal pattern is observed in Figure 4b. The velocity errors are concentrated primarily near the two boundaries, whereas the reconstruction remains relatively stable over the central observation interval. M9 achieves the lowest overall velocity RMSE. The consistent improvements in both position and velocity reconstruction indicate that the predicted systematic-error sequences effectively correct the observations without introducing additional temporal inconsistency into the reconstructed trajectory.

Among the one-shot model-driven methods M1–M4, M4 provides the best overall performance, indicating that spline regularization and parameter alignment can improve both trajectory reconstruction and systematic-error estimation. Nevertheless, B-spline coefficients and systematic-error parameters are still optimized within the same estimation problem. Consequently, slowly varying systematic-error responses may continue to compete with the flexible spline representation for explaining the same observation residuals.

The data-driven baselines M5–M8 show progressively lower trajectory-reconstruction errors and ${\mathrm{nRMSE}}_{U}$ within that group. However, trajectory accuracy and systematic-error-sequence recovery do not follow the same ranking across the model-driven and data-driven methods. For example, M5 attains a lower position RMSE than M4 (1.1757 m versus 1.2383 m) but a substantially larger ${\mathrm{nRMSE}}_{U}$ (8.5554 versus 5.2811). Thus, improved trajectory reconstruction does not by itself imply more accurate recovery of the underlying systematic-error sequence. This discrepancy between the two domains is consistent with partial absorption of structured residual components by the estimated trajectory.

M9’s advantage under matched conditions reflects the combined effect of its residual-driven compensation strategy and task-oriented network design. First, systematic errors are predicted in the observation domain before trajectory re-estimation, so the network-predicted error sequence and B-spline coefficients are not estimated as a single joint parametric correction within the same normal-equation system. Second, multichannel temporal modeling allows the network to exploit consistency across sensors, measurement types, and sampling epochs when separating persistent or slowly varying patterns from local random fluctuations. Third, sensor-identity and measurement-type embeddings provide channel-specific information beyond normalized residual amplitudes alone; the dependence on the fixed sensor–error association is examined separately in Section 4.4.3. Finally, the trajectory reconstruction loss constrains the predicted error sequence according to its downstream effect on position and velocity estimation rather than solely through pointwise observation-domain agreement.

Accordingly, the performance advantage of ResCompFormer is interpreted first in terms of the change in estimation strategy: systematic errors are predicted in the observation domain rather than jointly estimated with the B-spline coefficients, thereby avoiding their direct competition within the same parametric estimation problem. Within this decoupled formulation, temporal and cross-channel regularities learned from the training data further improve systematic-error prediction over the considered data distribution. This decoupling does not create additional algebraic information or alter the structural identifiability conditions of the original observation model; rather, it avoids the specific weak-identifiability mechanism introduced by joint parametric estimation in the B-spline-constrained EMBET formulation.

The cross-domain comparison should be interpreted by distinguishing parameter recovery from error-sequence recovery. A relatively large parameter-space error can coexist with a smaller observation-domain discrepancy because different parameter combinations may generate similar slowly varying responses, particularly along weakly sensitive directions; in addition, the component overlapping the spline sensitivity space may be partially absorbed by the trajectory correction.

### 4.4. Robustness Under Distribution, Model, and Sensor Variations

The experiments in Section 4.2 and Section 4.3 use the systematic-error family in Equation (92) for both data generation and the parametric model-driven estimators. To examine performance beyond this matched setting, the test conditions are varied in four ways: parameter-distribution shift, functional-form mismatch, randomized channel–error assignment, and sensor dropout. Unless otherwise stated, the neural-network parameters obtained from the baseline training and validation sets are kept fixed in these experiments.

#### 4.4.1. Parameter-Distribution Shift

Let the original sampling interval of parameter $\gamma_{a}$ in Table 3 be $\left[l_{a},u_{a}\right]$, with center $c_{a}=\left(l_{a}+u_{a}\right)/2$ and half-width $h_{a}=\left(u_{a}-l_{a}\right)/2$. The shifted test samples draw the parameter from $\left[c_{a}-1.5h_{a},c_{a}-h_{a}\right]\cup \left[c_{a}+h_{a},c_{a}+1.5h_{a}\right]$, while the training and validation sets retain the original intervals. Thus, the test parameters lie outside the training support without changing the error functional form. For M4, the numerical optimization bounds are expanded to include the shifted intervals. M9 is evaluated without retraining, so this experiment isolates parameter-distribution shift from functional-form mismatch.

#### 4.4.2. Unseen Systematic-Error Form

Functional-form mismatch is evaluated by replacing the saturating-exponential drifts in the corresponding channels of Table 2 with continuous piecewise-linear drifts, while the constant-bias and linear-drift channels remain unchanged. The piecewise-linear error is defined as

(99) $$u_{i,d}^{\mathrm{pw}}\left(t_{j}\right)=\left\{\begin{array}{cc} k_{1,i,d}\Delta t_{j}, & \Delta t_{j}\leq t_{c},\\ k_{1,i,d}t_{c}+k_{2,i,d}\left(\Delta t_{j}-t_{c}\right), & \Delta t_{j}>t_{c}. \end{array}\right.$$

The breakpoint and slopes are sampled so that the RMS magnitude of the resulting error sequence is comparable with that of the corresponding saturating-exponential error generated by Equation (92). M4 continues to use the parametric model in Equation (92), whereas M9 is evaluated without exposure to piecewise-linear errors during training.

#### 4.4.3. Randomized Channel–Error Assignment

To examine the dependence of the learned representation on the fixed association between sensor identity and systematic-error type in Table 2, the active error types are randomly permuted among compatible sensor channels for each trajectory while preserving the number of constant, linear, and exponential errors and their parameter ranges. In the “Fixed→Random” setting, M9 is trained using the fixed assignment in Table 2 and evaluated directly on randomized assignments. In the “Random→Random” setting, a second ResCompFormer is trained using independently randomized assignments and evaluated on new randomized assignments. The latter setting removes a persistent association between a specific sensor identity and a specific error type across trajectories.

#### 4.4.4. Dropout of Previously Known Sensors

The fixed channel ordering in Equation (55) is retained when evaluating sensor dropout. For each test trajectory, one, two, or three of the 11 known sensors are randomly selected as unavailable over the complete observation interval, corresponding to dropout ratios of approximately 9.1%, 18.2%, and 27.3%. The normalized residual entries of the corresponding channels are set to zero to preserve the network input dimension, and the same channels are assigned zero statistical weight during the subsequent EMBET trajectory re-estimation. The trained network is used without retraining. In this experiment, ${\mathrm{nRMSE}}_{U}$ is calculated only over the remaining available channels.

The results in Table 8 show that the tested methods respond differently to distribution and model variations. Under the parameter-distribution shift, both M4 and M9 exhibit increased errors, while M9 maintains lower errors in both trajectory reconstruction and observation-domain systematic-error estimation. The degradation of M4 becomes more pronounced under the piecewise-linear mismatch because the prescribed parametric error model no longer matches the data-generating process, whereas M9 also degrades but remains at a lower error level. Under randomized channel–error assignments, the Fixed→Random setting exhibits a clear performance degradation relative to the matched reference, indicating that the persistent sensor–error association contributes useful statistical information under the baseline training distribution. In contrast, Random→Random training recovers much of the lost accuracy even though no fixed one-to-one association between sensor identity and error type is retained across trajectories. This recovery indicates that, when assignment variability is represented during training, the model can rely more strongly on temporal residual structure, measurement-type information, and multichannel context rather than on a persistent identity–error association.

Table 9 shows a gradual increase in reconstruction error as additional known sensors become unavailable. With three of the 11 sensors removed, the position RMSE increases from 0.7577 m to 0.9856 m, and the velocity RMSE and ${\mathrm{nRMSE}}_{U,\mathrm{avail}}$ follow the same progressive trend. The absence of an abrupt failure indicates that masking unavailable channels while assigning them zero statistical weight allows the current architecture to accommodate the temporary loss of previously known sensors without changing the network input dimension. The remaining channels continue to provide useful information for compensation, although the increasing error and variability also show that the fixed-sensor representation becomes progressively less reliable as observation loss increases.

The behavior for newly introduced sensors is different from dropout of previously known sensors. The current architecture uses predefined sensor-identity embeddings in Equation (53) and a fixed-dimensional all-channel projection in Equation (56). A sensor identity that is absent from training has no learned identity embedding, and adding observation channels changes the input dimension and channel ordering assumed by $W_{f}$. Direct insertion of previously unseen sensors is therefore not supported by the present formulation. A variable-sensor extension would require representations derived from sensor attributes or observation geometry together with a permutation-aware or set-based channel aggregation mechanism, rather than a fixed identity lookup and fixed-dimensional concatenation.

Together, Table 8 and Table 9 characterize four departures from the baseline condition: parameter extrapolation, error–model mismatch, variation in the sensor–error association, and loss of previously available sensors. These tests complement the matched-condition comparison in Section 4.3 by characterizing how the estimation accuracy changes when the test data depart from the conditions represented in the original training set.

### 4.5. Ablation and Sensitivity Analysis

#### 4.5.1. Component Ablation

Four ablated variants are evaluated to quantify the contributions of the main ResCompFormer components. The complete model is denoted by RCF-Full.

The variants without sensor-identity embedding, measurement-type embedding, and trajectory reconstruction loss are denoted by RCF-NoSE, RCF-NoMTE, and RCF-NoTL, respectively. The encoder-only variant is denoted by RCF-Enc; in this variant, the Transformer decoder is removed and the systematic-error sequence is predicted directly from the encoder output. All variants use the same data partition, training settings, and iterative compensation procedure as RCF-Full.

As shown in Table 10, removing any of the examined components degrades ResCompFormer performance. This confirms that the sensor-identity embedding, measurement-type embedding, trajectory reconstruction loss, and Transformer decoder each contribute to the final estimation accuracy.

Removing the sensor-identity embedding increases the position RMSE, velocity RMSE, and systematic-error nRMSE, showing that sensor identity carries useful channel-specific information in the matched setting. This information can reflect differences in sensor location, observation geometry, and measurement characteristics, while the fixed baseline assignment also creates a persistent association between particular identities and error types. The randomized-assignment results in Table 8 distinguish these effects further: performance decreases when the fixed association is changed only at test time but largely recovers when assignment variability is also included during training.

Compared with RCF-NoSE, the RCF-NoMTE variant shows a larger degradation, particularly in systematic-error reconstruction. This result indicates that explicitly distinguishing measurement types is important because range, range-rate, azimuth, and elevation measurements retain distinct physical meanings and temporal characteristics even after channel-wise normalization.

Removing the trajectory reconstruction loss produces a larger relative increase in position and velocity RMSE than in systematic-error nRMSE. This pattern shows that an error sequence that closely matches the observation-domain target is not necessarily optimal for downstream trajectory reconstruction. The trajectory-consistency supervision therefore constrains the predicted systematic errors according to their effects on reconstructed position and velocity.

The encoder-only RCF-Enc variant shows the largest overall degradation among the ablated variants, indicating that the decoder is important for transforming multichannel residual features into temporally structured systematic-error sequences. Overall, the ablation results show that the four examined components make complementary contributions to systematic-error compensation and trajectory reconstruction.

#### 4.5.2. Sensitivity to Residual Normalization

The main ResCompFormer configuration normalizes each residual channel by the prescribed measurement-noise standard deviation $\sigma_{c}$, as defined in Equation (43). This scaling expresses residual magnitude relative to the random-noise level of each heterogeneous observation channel. Because the systematic-error component can exceed the corresponding random-noise standard deviation, the normalized residual magnitudes may exceed unity. To assess the sensitivity of the learned compensation mapping to this scaling choice, a training-set RMS normalization is used as an alternative.

For RCF-RMS, a fixed scale is computed separately for each observation channel *c* from the initial residuals of the training trajectories,

(100) $$s_{c}^{\mathrm{RMS}}=\sqrt{\frac{1}{N_{\mathrm{train}}m}\sum_{\ell =1}^{N_{\mathrm{train}}}\sum_{j=1}^{m}{\left(r_{c}^{\left(0,\ell \right)}\left(t_{j}\right)\right)}^{2}},$$

where $N_{\mathrm{train}}=800$ is the number of training trajectories and *m* is the number of sampling epochs. Here, the superscript (0,ℓ) denotes the initial residual associated with training trajectory *ℓ*. The corresponding network input is ${\bar{r}}_{c}^{\left(k\right)}\left(t_{j}\right)=r_{c}^{\left(k\right)}\left(t_{j}\right)/s_{c}^{\mathrm{RMS}}$. The RMS scale is computed from the training set only and is then held fixed for validation, testing, and all outer compensation iterations.

RCF-NoiseStd denotes the baseline M9 configuration using $\sigma_{c}$. Because the two normalization rules produce different input distributions, RCF-RMS is trained independently from scratch, while the data partition, network architecture, loss functions, optimizer, and iterative-compensation settings are kept unchanged. In both configurations, the network output remains the signed systematic-error sequence in the original observation units.

As shown in Table 11, RCF-RMS remains close to the baseline in all three metrics. Relative to RCF-NoiseStd, the mean position RMSE, velocity RMSE, and ${\mathrm{nRMSE}}_{U}$ increase by 3.9%, 2.6%, and 3.6%, respectively. These modest changes indicate that the compensation accuracy is not highly sensitive to the residual scaling rule within the tested setting.

Noise standard-deviation normalization nevertheless gives consistently lower errors and is therefore retained in the main model. In addition to its slightly better empirical performance, scaling by $\sigma_{c}$ has a direct physical interpretation because each residual is expressed relative to the prescribed random uncertainty of its channel. By contrast, the training-set RMS scale contains both random-noise and systematic-error energy and can therefore compress channels with larger structured residuals more strongly. The comparison supports noise standard-deviation normalization as a stable choice for the heterogeneous-noise setting considered here, without implying that it is universally optimal.

#### 4.5.3. Autoregressive Versus Parallel Decoding and Start-Token Length

ResCompFormer employs a non-autoregressive decoder to generate the predicted signed systematic-error values for all sampling epochs in parallel. For comparison, an autoregressive variant, denoted by RCF-AR, is constructed using the same encoder and a causal Transformer decoder with the same number of decoder layers, attention heads, and hidden dimension as the parallel decoder, denoted by RCF-NAR. A zero-valued start vector initializes the autoregressive decoder at the first sampling epoch; thereafter, the preceding *C*-dimensional systematic-error vector is projected to $d_{\mathrm{model}}$ and used as the decoder input for the next sampling epoch. Full teacher forcing is used during training, whereas the previously predicted systematic-error vector is recursively fed back during inference.

RCF-AR and RCF-NAR are trained independently from scratch using the same training and validation split, residual input, loss functions, optimizer settings, and model-selection criterion. They are evaluated on the same 100 matched-condition test trajectories and use the same iterative trajectory-re-estimation procedure. The reported standard deviations are calculated across the test trajectories.

With the sampling interval $T_{s}=0.1$ s and m=2001 sampling epochs, the baseline non-autoregressive model uses $L_{\mathrm{start}}=L_{0}=200$, corresponding to 20 s of explicit decoder context and approximately 10% of the complete sequence. Start-token sensitivity is evaluated at $L_{\mathrm{start}}\in \left\{100,200,400\right\}$, corresponding to 10, 20, and 40 s, respectively. These settings span approximately 5%, 10%, and 20% of the complete sequence. In all three cases, the decoder retains access to the complete encoder memory $H^{\left(N_{e}\right)}$; therefore, varying $L_{\mathrm{start}}$ changes the amount of explicit start-token context without removing the globally encoded residual information.

As shown in Table 12, the parallel baseline with $L_{\mathrm{start}}=200$ achieves the lowest mean errors among the tested decoder configurations. Relative to this baseline, RCF-AR increases the position RMSE, velocity RMSE, and ${\mathrm{nRMSE}}_{U}$ by approximately 10.1%, 9.1%, and 10.5%, respectively. These results indicate that recursive generation does not improve systematic-error compensation in the present long-sequence setting. The degradation is consistent with the mismatch between teacher-forced training and recursive inference, together with the propagation of prediction errors through recursive feedback.

Parallel decoding, however, does not imply temporally independent prediction. Temporal dependencies are already represented by the attention-based encoder–decoder architecture, and every prediction position can use the complete encoder memory without conditioning on previously predicted errors. This allows the systematic-error sequence to be generated jointly while avoiding recursive error propagation.

The start-token sensitivity results show a moderate dependence on explicit decoder context. Reducing $L_{\mathrm{start}}$ from 200 to 100 increases the three metrics by approximately 4.5%, 3.7%, and 4.7%, whereas increasing it from 200 to 400 increases them by only approximately 1.7%, 1.6%, and 1.6%, respectively, without improving accuracy. This pattern suggests that 200 sampling epochs provide sufficient start-token context for the present data, while a shorter segment loses useful context and a longer segment provides little additional benefit. Accordingly, $L_{\mathrm{start}}=200$ is retained as the default setting within the tested range.

### 4.6. Computational Efficiency and Accuracy–Runtime Trade-Off for Offline Trajectory Refinement

Computational efficiency is evaluated during the testing stage after the parameters of the data-driven models have been fixed. All methods are evaluated on the same workstation described in Section 4.3.2, equipped with an NVIDIA RTX 4070 GPU, an Intel Core i9-14900 processor, and 64 GB of memory. The reported total runtime denotes the end-to-end processing time for one complete trajectory, including residual construction, systematic-error estimation, observation compensation, B-spline/EMBET trajectory reconstruction, and all required optimization or iterative compensation procedures. For the neural-network methods, the network prediction time is additionally recorded to separate the computational cost of systematic-error prediction from that of the complete trajectory-refinement pipeline.

The number of trainable parameters is reported for the neural-network methods as a measure of model size. This quantity is not applicable to M1–M4, M10, and M11 because these methods estimate trajectory and systematic-error parameters directly for each observation record rather than using a set of learned network parameters. For the one-shot model-driven methods M1–M4, $N_{\mathrm{outer}}=1$. For the iterative methods, $N_{\mathrm{outer}}$ denotes the average number of compensation and trajectory re-estimation cycles required to satisfy the stopping criteria during testing.

As shown in Table 13, model size and prediction latency do not follow a strictly monotonic relationship across the neural-network methods because their computational costs also depend on the sequence representation and the degree of parallelism in the corresponding architectures. For example, M8 contains more trainable parameters than M7 but requires less prediction time. In M7, the Transformer encoder operates along the temporal dimension of the m=2001 sampling epochs, whereas M8 represents the C=22 observation channels as tokens and applies attention across the channel dimension. The shorter token sequence of M8 therefore reduces the attention-related computational cost despite its slightly larger parameter count.

M9 has approximately 27.57 million trainable parameters, substantially more than the other neural baselines, owing to its $d_{\mathrm{model}}=512$ representation and four-layer-encoder–three-layer-decoder architecture. Despite its larger model size, M9 requires 44.8 ms to predict one complete residual sequence. With an average of 3.026 outer iterations, the accumulated network-prediction time is approximately 0.136 s per trajectory, which represents only about 1.3% of the 10.536 s end-to-end processing time. Thus, most of the computational cost of the complete M9 procedure arises from the repeated observation compensation and B-spline/EMBET trajectory re-estimation rather than from the ResCompFormer forward pass itself.

The comparison between parallel and autoregressive decoding further illustrates the influence of the inference strategy on computational efficiency. RCF-AR uses the same encoder–decoder dimensions as M9 and consequently has a comparable number of trainable parameters. However, the autoregressive decoder generates the systematic-error sequence sequentially, with each predicted *C*-dimensional error vector used to construct the input for the next sampling epoch. Its network prediction time therefore increases to approximately 5.86 s, compared with 44.8 ms for the parallel decoder of M9. With an average of 3.183 outer iterations, the accumulated network-prediction time of RCF-AR is approximately 18.66 s, accounting for a substantial portion of its 32.756 s end-to-end runtime. The marked latency difference between the two variants is therefore mainly associated with sequential autoregressive generation rather than with their model sizes.

The model-driven methods exhibit a different computational pattern. Among the one-shot methods, M1 has the lowest runtime, while M2–M4 require additional computation for their respective spline adaptation, regularization, and systematic-error estimation procedures. Introducing iterative refinement further increases the computational burden of the model-driven approach. M10 and M11 require 48.672 s and 63.418 s per trajectory, respectively, because systematic errors and trajectory parameters are repeatedly estimated over several compensation cycles. Although these iterative strategies improve the reconstruction performance of M4, their increased numerical optimization cost is considerably greater than that of M9.

Overall, M9 requires 10.536 s to process one complete trajectory and achieves the lowest position RMSE among the compared methods. Its computational cost is higher than those of the lightweight neural baselines M5–M8 but remains lower than those of the iterative model-driven methods M10 and M11 and the autoregressive RCF-AR variant. These results indicate that the parallel residual-driven compensation strategy provides a favorable balance between trajectory reconstruction accuracy and computational cost in the considered offline processing setting.

The proposed framework is intended for offline trajectory refinement after a complete multi-sensor observation arc has been collected, rather than for sample-by-sample real-time trajectory estimation. The current procedure uses residual information from the complete observation sequence and performs iterative batch compensation followed by B-spline/EMBET trajectory re-estimation. Consequently, its primary objective is to improve systematic-error compensation and trajectory reconstruction accuracy rather than to minimize online processing latency. The measured computational cost is therefore interpreted as the processing overhead required for high-accuracy post-processing of a complete trajectory record. Further development toward online operation would require a causal or streaming sequence-processing mechanism together with a more computationally efficient trajectory re-estimation strategy.

## 5. Conclusions

This study investigated the absorption of sensor systematic errors by flexible B-spline trajectory representations in multi-sensor trajectory reconstruction. The sensitivity-space analysis showed that the observation-domain responses of slowly varying systematic errors may overlap with the spline sensitivity space and consequently be partially absorbed into the spline-coefficient correction, thereby weakening the identifiability of the systematic-error parameters. To address this problem, a residual-driven ResCompFormer framework was developed to predict multichannel systematic-error sequences from observation residuals and iteratively refine the reconstructed trajectory. The simulation results show that ResCompFormer achieves more accurate systematic-error compensation and position and velocity reconstruction than the considered model-driven and data-driven methods. Among the model-driven variants, iterative compensation and stepwise parameter estimation progressively improve trajectory reconstruction and parameter recovery, while the remaining performance gap between M11 and ResCompFormer indicates that the advantage of the proposed method is not attributable to the iterative estimation schedule alone. Additional experiments further demonstrate the robustness of the proposed framework to variations in systematic-error characteristics and sensor availability.

Several limitations of the present study should nevertheless be acknowledged. The proposed framework avoids the weak-identifiability mechanism associated with jointly estimating B-spline coefficients and systematic-error parameters by replacing parametric systematic-error estimation with data-driven observation-domain error prediction. However, this change in estimation strategy does not alter the underlying algebraic identifiability conditions of the original observation model, and the accuracy of the predicted systematic errors remains dependent on the information contained in the residuals and on the data distribution represented during training. In addition, the current implementation assumes a predefined set and ordering of observation channels. Although the sensor-dropout experiments show gradual degradation when previously known sensors become unavailable, sensors absent during training cannot be directly incorporated without adapting the corresponding sensor representations and channel-processing modules. Finally, the iterative combination of systematic-error prediction and B-spline trajectory re-estimation makes the current framework more suitable for offline trajectory refinement than for sample-by-sample real-time estimation.

Future work will focus on systematic-error separation, sensor-configuration flexibility, and computational efficiency. Physics-informed constraints and uncertainty-aware priors will be investigated to improve robustness under more challenging weak-identifiability conditions. Flexible sensor representations and variable-set channel aggregation will be developed to accommodate changing sensor configurations and previously unseen sensors. More efficient sequence models and trajectory re-estimation algorithms, together with causal and streaming implementations, will also be explored to extend the current offline framework toward near-real-time and online multi-sensor trajectory reconstruction.

## Figures and Tables

**Table 1:** Simulation parameter settings for the UAV trajectory dataset.

Parameter	Value
Number of sensors *n*	11
Total number of observation channels *C*	22
Number of generated trajectories $N_{\mathrm{traj}}$	1000
Sampling interval $T_{s}$	0.1 s
Simulation duration $T_{\max}$	200 s
Number of sampling instants *m*	2001
Local north-coordinate range	[−5000,5000] m
Local east-coordinate range	[−5000,5000] m
Altitude range	[2000,4000] m
Flight-speed range	[35,90] m/s
Maximum nominal bank angle	$35^{\circ }$
Range-noise standard deviation $\sigma_{R}$	5 m
Range-rate-noise standard deviation $\sigma_{\dot{R}}$	0.3 m/s
Azimuth-noise standard deviation $\sigma_{A}$	$5^{\prime \prime }$
Elevation-noise standard deviation $\sigma_{E}$	$5^{\prime \prime }$

**Table 2:** Channel-wise systematic-error type configuration for the heterogeneous multi-sensor system.

Sensor	Radar Measurements		Optical Measurements	
	R	$\dot{R}$	A	E
$S_{1}$	Constant bias	None	–	–
$S_{2}$	Linear drift	None	–	–
$S_{3}$	Exponential drift	None	–	–
$S_{4}$	None	Constant bias	–	–
$S_{5}$	None	Linear drift	–	–
$S_{6}$	None	Exponential drift	–	–
$S_{7}$	None	None	–	–
$S_{8}$	–	–	Constant bias	None
$S_{9}$	–	–	Linear drift	Constant bias
$S_{10}$	–	–	Exponential drift	Linear drift
$S_{11}$	–	–	None	Exponential drift

**Table 3:** Nonzero systematic-error parameters used in the weak-identifiability and compensation experiments.

Sensor/Channel	Parameter	Fixed Value (Weak-Identifiability)	Sampling Range (Compensation)
**Constant-bias errors **			
$S_{1}/R$	$u_{1,R}^{0}$	20m	U(−30,30)m
$S_{4}/\dot{R}$	$u_{4,\dot{R}}^{0}$	1.2m/s	U(−1.8,1.8)m/s
$S_{8}/A$	$u_{8,A}^{0}$	20arcsec	U(−30,30)arcsec
$S_{9}/E$	$u_{9,E}^{0}$	20arcsec	U(−30,30)arcsec
**Linear-drift errors**			
$S_{2}/R$	$k_{2,R}$	0.173m/s	U(−0.260,0.260)m/s
$S_{5}/\dot{R}$	$k_{5,\dot{R}}$	$1.04\times 10^{-2}\;m/s^{2}$	$U\left(-1.56,1.56\right)\times 10^{-2}\;m/s^{2}$
$S_{9}/A$	$k_{9,A}$	0.173arcsec/s	U(−0.260,0.260)arcsec/s
$S_{10}/E$	$k_{10,E}$	0.173arcsec/s	U(−0.260,0.260)arcsec/s
**Saturating exponential-drift errors**			
$S_{3}/R$	$\beta_{3,R}$	25.1m	U(−37.5,37.5)m
	$\tau_{3,R}^{c}$	50s	U(30,70)s
$S_{6}/\dot{R}$	$\beta_{6,\dot{R}}$	−1.51m/s	U(−2.25,2.25)m/s
	$\tau_{6,\dot{R}}^{c}$	50s	U(30,70)s
$S_{10}/A$	$\beta_{10,A}$	25.1arcsec	U(−37.5,37.5)arcsec
	$\tau_{10,A}^{c}$	50s	U(30,70)s
$S_{11}/E$	$\beta_{11,E}$	−25.1arcsec	U(−37.5,37.5)arcsec
	$\tau_{11,E}^{c}$	50s	U(30,70)s

**Table 4:** Statistical results of the weak-identifiability experiment.

Metric	Mean	Std.	Min.	Max.
Noise-only position RMSE (m)	2.0738	0.3264	1.3186	3.2147
Joint-estimation position RMSE (m)	2.4637	0.4389	1.4269	3.9821
Noise-only velocity RMSE (m/s)	0.5739	0.0718	0.3987	0.8264
Joint-estimation velocity RMSE (m/s)	0.6216	0.0835	0.4213	0.9278
${\mathrm{sNRMSE}}_{\gamma }$	0.4236	0.2187	0.0946	1.2478
Systematic-response absorption $\eta_{U}$ (%)	26.4187	0.0024	26.4153	26.4221

**Table 5:** Estimation statistics of representative systematic-error parameters in the weak-identifiability experiment.

Sensor/ Channel	Parameter (Unit)	True Value	Estimated Value Mean ± Std.	Mean Absolute Normalized Error
$S_{1}/R$	$u_{1,R}^{0}$ (m)	20.0000	20.0186±0.4217	0.0168
$S_{2}/R$	$k_{2,R}$ (m/s)	0.1730	0.172314±0.011528	0.0533
$S_{3}/R$	$\beta_{3,R}$ (m)	25.1000	27.1846±7.2368	0.2395
$S_{3}/R$	$\tau_{3,R}^{c}$ (s)	50.0000	56.4827±14.8365	0.2864
$S_{4}/\dot{R}$	$u_{4,\dot{R}}^{0}$ (m/s)	1.2000	1.19891±0.02314	0.0154
$S_{5}/\dot{R}$	$k_{5,\dot{R}}$ (m/s^2^)	0.0104	0.010431±0.000676	0.0519
$S_{6}/\dot{R}$	$\beta_{6,\dot{R}}$ (m/s)	−1.5100	−1.64218±0.45173	0.2488
$S_{6}/\dot{R}$	$\tau_{6,\dot{R}}^{c}$ (s)	50.0000	57.1364±16.2478	0.3176
$S_{8}/A$	$u_{8,A}^{0}$ (arcsec)	20.0000	20.0127±0.5086	0.0203
$S_{9}/A$	$k_{9,A}$ (arcsec/s)	0.1730	0.173384±0.013742	0.0634
$S_{10}/A$	$\beta_{10,A}$ (arcsec)	25.1000	28.5364±9.3187	0.3161
$S_{10}/A$	$\tau_{10,A}^{c}$ (s)	50.0000	59.2741±18.6352	0.3897
$S_{11}/E$	$\beta_{11,E}$ (arcsec)	−25.1000	−28.2471±9.0476	0.3048
$S_{11}/E$	$\tau_{11,E}^{c}$ (s)	50.0000	58.8163±17.9246	0.3715

**Table 6:** Overall comparison of trajectory reconstruction and systematic-error estimation performance.

Method	Position RMSE (m)	Velocity RMSE (m/s)	${\mathrm{nRMSE}}_{U}$
M1	2.4976±0.4128	0.6283±0.0936	5.4550±0.8641
M2	1.8077±0.3055	0.4093±0.0714	5.4448±0.8217
M3	1.3064±0.2219	0.3658±0.0603	5.4487±0.7965
M4	1.2383±0.1986	0.3278±0.0517	5.2811±0.7338
M5	1.1757±0.2142	0.3293±0.0548	8.5554±1.2473
M6	1.0973±0.1895	0.3073±0.0496	7.2753±1.0642
M7	1.0190±0.1708	0.2854±0.0442	6.0965±0.8831
M8	0.9144±0.1469	0.2561±0.0387	4.8797±0.6924
M9	0.7577±0.1184	0.2122±0.0315	3.5531±0.5089
M10	1.1546±0.1863	0.3097±0.0491	5.0124±0.7095
M11	1.0829±0.1761	0.2916±0.0462	4.7318±0.6742

*Note:* Bold values indicate the best performance for each evaluation metric.

**Table 7:** Range-normalized parameter-error metrics of the model-driven methods.

Method/Setting	${\mathrm{pNRMSE}}_{\gamma }$	${\mathrm{pBias}}_{\gamma }$	${\mathrm{pMAE}}_{\gamma }$
M1	0.356	0.012	0.181
M2	0.321	0.008	0.164
M3	0.294	−0.006	0.151
M4	0.268	0.005	0.137
M10	0.243	0.004	0.124
M11	0.216	0.003	0.109

*Note:* Bold values indicate the lowest pNRMSE and pMAE among the compared methods.

**Table 8:** Robustness under parameter-distribution shift, functional–form mismatch, and randomized channel–error assignment.

Test Condition	Method	Position RMSE (m)	Velocity RMSE (m/s)	${\mathrm{nRMSE}}_{U}$
Matched reference	M4	1.2383±0.1986	0.3278±0.0517	5.2811±0.7338
Matched reference	M9	0.7577±0.1184	0.2122±0.0315	3.5531±0.5089
Parameter shift	M4	1.3146±0.2237	0.3459±0.0578	5.5173±0.8062
Parameter shift	M9	0.8612±0.1398	0.2367±0.0374	4.0186±0.5927
Piecewise mismatch	M4	1.5634±0.2789	0.4046±0.0708	6.4728±0.9725
Piecewise mismatch	M9	0.9179±0.1573	0.2497±0.0408	4.2145±0.6538
Random assignment	M9 Fixed→Random	1.0189±0.1815	0.2784±0.0473	4.7265±0.7421
Random assignment	M9 Random→Random	0.8297±0.1372	0.2281±0.0358	3.8619±0.5654

*Note:* Bold values indicate the best performance under each test condition.

**Table 9:** Sensitivity of the trained ResCompFormer to dropout of previously known sensors.

Dropped Sensors	Position RMSE (m)	Velocity RMSE (m/s)	${\mathrm{nRMSE}}_{U,\mathrm{avail}}$
0/11	0.7577±0.1184	0.2122±0.0315	3.5531±0.5089
1/11	0.8048±0.1315	0.2251±0.0348	3.6816±0.5427
2/11	0.8847±0.1492	0.2463±0.0398	3.9158±0.6021
3/11	0.9856±0.1736	0.2738±0.0462	4.2879±0.6814

*Note:* Bold values indicate the best performance for each evaluation metric across the sensor-dropout settings.

**Table 10:** Ablation results of ResCompFormer on the test set.

Variant	Position RMSE (m)	Velocity RMSE (m/s)	${\mathrm{nRMSE}}_{U}$
RCF-Full	0.7577±0.1184	0.2122±0.0315	3.5531±0.5089
RCF-NoSE	0.8259±0.1276	0.2292±0.0342	3.9440±0.5668
RCF-NoMTE	0.8562±0.1328	0.2377±0.0354	4.1572±0.5975
RCF-NoTL	0.8941±0.1407	0.2483±0.0369	3.7663±0.5416
RCF-Enc	0.9395±0.1494	0.2589±0.0388	4.4414±0.6387

*Note:* Bold values indicate the best performance among the full model and its ablated variants.

**Table 11:** Sensitivity to residual normalization on the matched-condition test set.

Normalization	Position RMSE (m)	Velocity RMSE (m/s)	${\mathrm{nRMSE}}_{U}$
RCF-NoiseStd (baseline)	0.7577±0.1184	0.2122±0.0315	3.5531±0.5089
RCF-RMS	0.7869±0.1236	0.2178±0.0324	3.6826±0.5261

*Note:* Bold values indicate the best performance among the compared normalization schemes.

**Table 12:** Decoder -strategy comparison and start-token-length sensitivity on the matched-condition test set.

Variant	$L_{\mathrm{start}}$	Position RMSE (m)	Velocity RMSE (m/s)	${\mathrm{nRMSE}}_{U}$
RCF-AR	–	0.8346±0.1379	0.2316±0.0360	3.9264±0.5812
RCF-NAR	100	0.7918±0.1267	0.2201±0.0331	3.7216±0.5407
RCF-NAR (baseline)	200	0.7577±0.1184	0.2122±0.0315	3.5531±0.5089
RCF-NAR	400	0.7705±0.1219	0.2155±0.0322	3.6108±0.5168

*Note:* Bold values indicate the best performance among the tested decoder configurations and start-token lengths.

**Table 13:** Computational efficiency, model size, and trajectory reconstruction accuracy of the compared methods. The total runtime denotes the end-to-end processing time for one complete trajectory.

Method	Trainable Parameters (M)	Network Prediction (ms)	$N_{\mathrm{outer}}$	Total Runtime (s/trajectory)	Position RMSE (m)
M1	–	–	1	3.842	2.4976
M2	–	–	1	9.637	1.8077
M3	–	–	1	12.416	1.3064
M4	–	–	1	15.783	1.2383
M5	2.16	19	3.912	7.864	1.1757
M6	1.34	6	3.684	7.213	1.0973
M7	3.17	32	3.437	8.647	1.0190
M8	4.19	14	3.218	9.284	0.9144
M9	27.57	45	3.026	10.536	0.7577
M10	–	–	3.146	48.672	1.1546
M11	–	–	3.428	63.418	1.0829
RCF-AR	27.47	5862	3.183	32.756	0.8346

## Data Availability

The simulation datasets generated and analyzed in this study are available from the corresponding author upon reasonable request. The data-generation procedure and the main simulation settings are described in Section 4 to facilitate the reproduction of the experiments.

## Abbreviations

The following abbreviations are used in this manuscript:

6-DOF	Six degrees of freedom
BIC	Bayesian information criterion
BiLSTM	Bidirectional long short-term memory
ECEF	Earth-centered, Earth-fixed
EMBET	Error Model Best Estimate of Trajectory
ENU	East–north–up
FC	Fully connected layer
FFN	Feed-forward network
GELU	Gaussian error linear unit
GPU	Graphics processing unit
IMU	Inertial measurement unit
MEMS	Microelectromechanical systems
MHA	Multi-head attention
NED	North–east–down
nRMSE	Noise-normalized root mean square error
pBias	Signed range-normalized parameter bias
pMAE	Range-normalized parameter mean absolute error
pNRMSE	Range-normalized parameter root mean square error
RCF	ResCompFormer
RCF-AR	Autoregressive ResCompFormer variant
RCF-NAR	Non-autoregressive ResCompFormer variant
RMSE	Root mean square error
sNRMSE	Scale-normalized root mean square error
TCN	Temporal convolutional network
UAV	Unmanned aerial vehicle

## Appendix A. Notation and Symbol Definitions

The principal mathematical symbols used throughout the manuscript are summarized in Table A1. Symbols used only locally in individual derivations are defined at their first occurrence in the main text.

**Table A1:** Principal mathematical symbols used in the manuscript.

Symbol	Definition
$t_{j}$	The *j*th sampling epoch.
*m*	Number of sampling epochs in one observation trajectory.
*n*	Number of sensors in the heterogeneous sensor system.
D, $D_{i}$	Candidate set of measurement types and the subset available from sensor *i*, respectively.
*C*	Total number of available observation channels, with each channel corresponding to a sensor–measurement-component pair.
Ω	Set of all available observation channels in the heterogeneous sensor system.
x(t), $\dot{x}\left(t\right)$	Target position and velocity in the ECEF frame at time *t*, respectively.
α(t)	Target state vector containing position and velocity at time *t*.
X	Target-state vector obtained by stacking the states over all sampling epochs.
Y	Global observation vector obtained by stacking all available multi-sensor measurements.
F(·)	Nonlinear observation mapping from the target trajectory representation to the observation domain.
U(t;γ)	Signed systematic-error vector in the observation domain, parameterized by γ.
ϵ	Random measurement-noise vector.
Σ	Covariance matrix of the random measurement noise.
P	Weighting matrix used in weighted least-squares estimation, with $P=\Sigma^{-1}$.
$\sigma_{i,d}$, $\sigma_{c}$	Measurement-noise standard deviation for measurement component *d* of sensor *i* and its equivalent channel-indexed notation, respectively.
*p*	B-spline degree; the corresponding basis-function order is p+1.
*q*	Number of B-spline basis functions, q=N+p+1, where *N* denotes the number of interior knots.
T	Extended B-spline knot vector.
$B_{\tau ,p+1}\left(t\right)$	B-spline basis function of order p+1 associated with basis index τ.
B(t), $B_{s}$	B-spline state-mapping matrix at time *t* and its stacked form over all sampling epochs, respectively.
b	B-spline coefficient vector used to represent the target trajectory.
γ	Parameter vector of the prescribed systematic-error model.
$n_{\gamma }$	Dimension of the full prescribed systematic-error parameter vector γ.
$N_{\gamma }$	Number of active systematic-error parameters included in the parameter-domain evaluation metrics.
$u_{i,d}^{0}$, $k_{i,d}$, $\beta_{i,d}$, $\tau_{i,d}^{c}$	Constant-bias coefficient, linear-drift coefficient, saturating-exponential magnitude, and corresponding time constant for measurement component *d* of sensor *i*, respectively.
$J_{b_{k}}$, $J_{\gamma_{k}}$	Sensitivity matrices with respect to the B-spline coefficients and systematic-error parameters at iteration *k*, respectively.
$\Pi_{b_{k}}$	P-weighted orthogonal projector onto the spline sensitivity space.
$M_{b_{k}}$	Complementary projection matrix of the spline sensitivity space, defined as $I-\Pi_{b_{k}}$.
$S_{\gamma_{k}}$	Profiled Schur-complement information matrix associated with the systematic-error parameters.
$R_{k}$, ${\bar{R}}_{k}$	Stacked observation-residual vector and the corresponding noise-standardized residual matrix at compensation iteration *k*, respectively.
$N_{\mathrm{obs}}$	Number of observation elements in one complete trajectory, $N_{\mathrm{obs}}=mC$.
$N_{\mathrm{test}}$	Number of trajectories in the test set used to summarize evaluation metrics.
$d_{e}$	Dimension of the residual, sensor-identity, and measurement-type embeddings.
$d_{\mathrm{model}}$	Hidden feature dimension of the Transformer encoder and decoder.
$N_{e}$, $N_{d}$, $N_{h}$	Numbers of encoder layers, decoder layers, and attention heads, respectively.
θ, $\theta^{*}$	Trainable ResCompFormer parameters and their optimized values, respectively.
$L_{\mathrm{start}}$	Length of the encoded residual feature segment used as the decoder start-token sequence.
${\tilde{U}}_{k}$, ${\hat{U}}_{k}$	Raw ResCompFormer systematic-error prediction and the damped systematic-error estimate used for observation compensation, respectively.
η	Damping factor used in the iterative systematic-error update.
$\eta_{U}$	Absorption ratio of the true systematic-error response projected onto the spline sensitivity space.
$\tau_{b}$, $\tau_{U}$	Convergence thresholds for the B-spline coefficients and systematic-error estimate, respectively.
$L_{e}$, $L_{t}$	Systematic-error prediction loss and trajectory reconstruction loss, respectively.
$\lambda_{1}$, $\lambda_{2}$	Weighting coefficients of the systematic-error prediction and trajectory reconstruction losses, respectively.
$\alpha_{x}$, $\alpha_{\dot{x}}$	Relative weights of the position and velocity terms in the trajectory reconstruction loss.
$\sigma_{x}$, $\sigma_{\dot{x}}$	Fixed normalization scales for position and velocity reconstruction errors in the trajectory loss.
L	Total training loss formed by combining the systematic-error prediction and trajectory reconstruction losses.
